# Assessment of Pretreatment With Oral P2Y12 Inhibitors and Cardiovascular and Bleeding Outcomes in Patients With Non-ST Elevation Acute Coronary Syndromes: A Systematic Review and Meta-analysis

**DOI:** 10.1001/jamanetworkopen.2021.34322

**Published:** 2021-11-19

**Authors:** Luke P. Dawson, David Chen, Misha Dagan, Jason Bloom, Andrew Taylor, Stephen J. Duffy, James Shaw, Jeffrey Lefkovits, Dion Stub

**Affiliations:** 1 Department of Cardiology, The Royal Melbourne Hospital, Melbourne, Victoria, Australia; 2 Department of Epidemiology and Preventive Medicine, Monash University, Melbourne, Victoria, Australia; 3 Department of Cardiology, The Alfred Hospital, Melbourne, Victoria, Australia; 4 The Baker Institute, Melbourne, Victoria, Australia

## Abstract

**Importance:**

The practice of pretreatment with oral P2Y12 inhibitors in non-ST elevation acute coronary syndromes (NSTEACS) remains common; however, its association with improved cardiovascular outcomes is unclear.

**Objective:**

To assess the association between oral P2Y12 inhibitor pretreatment and cardiovascular and bleeding outcomes in patients with NSTEACS.

**Data Sources:**

On March 20, 2021, PubMed, MEDLINE, Embase, Scopus, Web of Science, Science Direct, clinicaltrials.gov, and the Cochrane Central Register for Controlled Trials were searched from database inception.

**Study Selection:**

Randomized clinical trials of patients with NSTEACS randomized to either oral P2Y12 inhibitor pretreatment (defined as prior to angiography) or no pretreatment (defined as following angiography, once coronary anatomy was known) among patients undergoing an invasive strategy.

**Data Extraction and Synthesis:**

This study followed Preferred Reporting Items for Systematic Reviews and Meta-analyses guidelines. Data on publication year, sample size, clinical characteristics, revascularization strategy, P2Y12 inhibitor type and dosage, time from pretreatment to angiography, and end point data were independently extracted by 2 authors. A random-effects model was used, including stratification by (1) P2Y12 inhibitor type, (2) revascularization strategy, and (3) access site.

**Main Outcomes and Measures:**

The primary end point was 30-day major adverse cardiac events (MACEs). Secondary end points were 30-day myocardial infarction (MI) and cardiovascular death. The primary safety end point was 30-day major bleeding (defined according to individual studies).

**Results:**

A total of 7 trials randomizing 13 226 patients to either pretreatment (6603 patients) or no pretreatment (6623 patients) were included. The mean age of patients was 64 years and 3598 (27.2%) were female individuals. Indication for P2Y12 inhibitors was non-ST elevation myocardial infarction in 7430 patients (61.7%), radial access was used in 4295 (32.6%), and 10 945 (82.8%) underwent percutaneous coronary intervention. Pretreatment was not associated with a reduction in 30-day MACE (odds ratio [OR], 0.95; 95% CI, 0.78-1.15; *I*^2^ = 28%), 30-day MI (OR, 0.90; 95% CI, 0.72-1.12; *I*^2^ = 19%), or 30-day cardiovascular death (OR, 0.79; 95% CI, 0.49-1.27; *I*^2^ = 0%). The risk of 30-day major bleeding was increased among patients who underwent pretreatment (OR, 1.51; 95% CI, 1.16-1.97; *I*^2^ = 41%). The number needed to harm to bring about 1 major bleeding event with oral P2Y12 inhibitor pretreatment was 63 patients.

**Conclusions and Relevance:**

In this study, pretreatment with oral P2Y12 inhibitors among patients with NSTEACS prior to angiography, compared with treatment once coronary anatomy is known, was associated with increased bleeding risk and no difference in cardiovascular outcomes. Routine pretreatment with oral P2Y12 inhibitors in patients with NSTEACS receiving an early invasive strategy is not supported by this study.

## Key Points

**Question:**

What is the association of oral P2Y12 inhibitor pretreatment with cardiovascular and bleeding outcomes in patients with non-ST elevation acute coronary syndromes (NSTEACS)?

**Findings:**

In this systematic review and meta-analysis of 7 randomized clinical trials with 13 226 patients, oral P2Y12 pretreatment was associated with no difference in 30-day cardiovascular events, myocardial infarction, or cardiovascular death, but it was associated with an increased risk of bleeding.

**Meaning:**

In this study, pretreatment with oral P2Y12 inhibitors among patients with NSTEACS prior to angiography, compared with treatment once coronary anatomy is known, was associated with increased bleeding risk and no difference in cardiovascular outcomes.

## Teaser

This systematic review and meta-analysis assesses the association between oral P2Y12 inhibitor pretreatment and cardiovascular and bleeding outcomes in patients with non-ST elevation acute coronary syndromes.

## Introduction

The timing of oral P2Y12 inhibitor administration in patients with non-ST elevation acute coronary syndromes (NSTEACS) has been a matter of substantial debate over the last 2 decades.^1,2,3,4^ Pretreatment, defined as oral P2Y12 inhibitor administration prior to angiography and usually at the time of diagnosis, has been commonly used with the rationale that there may be greater platelet inhibition at time of percutaneous coronary intervention (PCI); there may be reduced ischemic events while awaiting angiography; and more potent antiplatelet agents, such as glycoprotein IIb/IIIa inhibitors, may be avoided.^4^ While these reasons may be logical, there are limited data to support this approach. Previous guidelines supporting a pretreatment strategy relied mostly on older and indirect data,^5,6^ and the most recent comprehensive meta-analysis (performed in 2014), which did not support pretreatment, relied mostly on observational rather than randomized data.^1,7^ Arguments against pretreatment mainly relate to increased bleeding risk among patients who require PCI, in addition to those who need coronary artery bypass grafting (in whom surgery may be delayed with increased risk of postoperative bleeding) and those who may have an alternate diagnosis (eg, aortic dissection). European guidelines recently changed to recommend against pretreatment in patients with NSTEACS based on several trials and observational studies.^8,9,10,11,12^ However, debate still remains regarding the optimal approach,^13^ and several further trials have been published that allow for a more comprehensive pooled systematic review.

The aim of this systematic review and meta-analysis was to assess the association of oral P2Y12 inhibitor pretreatment with cardiovascular and bleeding outcomes, using randomized data. Furthermore, we stratified our analysis by P2Y12 inhibitor type, revascularization strategy, and arterial access location.

## Methods

This article has been prepared in accordance with the Preferred Reporting Items for Systematic Reviews and Meta-analyses (PRISMA) reporting guideline.^14^ The protocol has been uploaded to the Prospective Register of Systematic Reviews (PROSPERO; CRD42021243362).

### Search Strategy

On March 20, 2021, we performed a systematic search of MEDLINE, PubMed, Embase, Scopus, Web of Science, ScienceDirect, clinicaltrials.gov, and the Cochrane Central Register for Controlled Trials. Following removal of duplicates, 2 authors (L.P.D. and D.C.) screened titles and abstracts and independently assessed full-text articles for inclusion based on prespecified criteria. Discrepancies were resolved through consensus. Additionally, we used backward snowballing (ie, review of references) to identify relevant texts from articles identified in the original search. The complete search strategy is presented in eTable 1 in the Supplement.

### Selection Criteria

Studies were considered eligible for inclusion in the primary analysis according to 5 criteria. The criteria were if they (1) were a randomized clinical trial (RCT); (2) enrolled participants with NSTEACS; (3) compared oral P2Y12 inhibitor administration prior to angiography (ie, pretreatment) with treatment following angiography once coronary anatomy was known (ie, no pretreatment); (4) presented data regarding any of the prespecified primary or secondary end points as detailed later; and (5) were published in English.

### Data Extraction

Data were extracted independently by 2 authors (L.P.D. and D.C.) for each study using a standardized study form to determine authors, year of publication, inclusion and exclusion criteria, sample size, baseline patient characteristics, arterial access location for angiography, revascularization approach (including percentage of patients who underwent PCI), P2Y12 inhibitor type and dosage, mean or median time from pretreatment loading dose until angiography in the pretreatment group, end point data, and end point definitions. Risk of bias was assessed independently by 2 investigators (L.P.D. and M.D.) using the revised Cochrane risk-of-bias tool (RoB 2).^15^

### End Points

The prespecified primary end point was major adverse cardiovascular events (MACEs). Key secondary end points were myocardial infarction (MI) and cardiovascular death. The primary safety end point was major bleeding. End point definitions for each included study are shown in eTable 2 in the Supplement, and end point data were extracted with the aim of maintaining consistency in definitions between studies. The preferred time for end point assessment was 30 days. If 30-day outcomes were not presented by the study, the nearest time point to 30 days was used.

### Statistical Analysis

Odds ratios (ORs) and 95% CIs were determined using the restricted maximum-likelihood random-effects model, with the estimate of heterogeneity determined using the Mantel-Haenszel method. The presence of heterogeneity was measured with Cochran *Q* test with a *P* < .10 considered significant. To measure consistency, the *I*^2^ test was used (with a value greater than 50% indicating high heterogeneity). For significant results in the primary analysis, numbers needed to treat and to harm were calculated by dividing 1 by the absolute risk increase or absolute risk reduction, respectively. Publication bias was assessed for the primary end point, secondary end points, and primary safety end point using Egger tests and funnel plots. Three prespecified subgroup analyses were performed for the primary end point and primary safety end point according to (1) the type of P2Y12 inhibitor used for pretreatment, (2) the revascularization strategy used (ie, no PCI or PCI), and (3) arterial access site (radial or femoral). To assess for any interaction between outcomes and time from oral P2Y12 inhibitor pretreatment to angiography, we performed a metaregression analysis for the primary end point and the primary safety end point using a random-effects model reporting metaregression coefficients and *P* values. The metaregression was performed first with all studies included, and second with the PCI Clopidogrel in Unstable Angina to Prevent Recurrent Events (PCI CURE) trial excluded given its outlier status (median 6-day period from pretreatment to angiography). Prespecified leave-one-out sensitivity analyses were performed for the primary end point and primary safety end point by iteratively removing 1 study at a time to ensure the findings were not reliant on a single study. Post hoc sensitivity analyses included performing the P2Y12 inhibitor subgroup analysis with the exclusion of the PCI CURE trial given that patients did not necessarily receive dual antiplatelets for the 30-day period after PCI and some received ticlopidine. All analyses were performed with R version 3.6.2 (R Project for Statistical Computing) using the meta (version 4.18-0) and metafor (version 2.4-0) packages. Statistical significance was set at *P* < .05, and all tests were 2-tailed.

## Results

**Figure 1.**
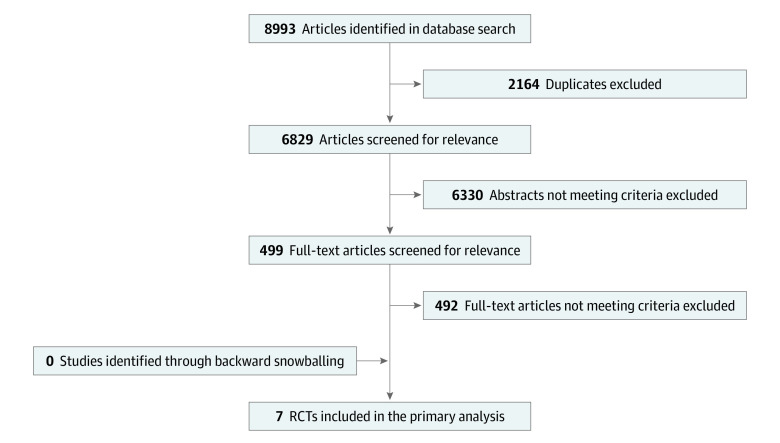
Study Selection RCT indicates randomized clinical trial.

The systematic search identified 8993 citations, of which 7 RCTs were included in the primary analysis (Figure 1).^5,9,11,16,17,18,19^ A total of 13 226 patients were included in the analysis, with 6603 patients randomly assigned to oral P2Y12 inhibitor pretreatment and 6623 patients randomly assigned to no pretreatment. Patient characteristics of included studies are presented in Table 1. Mean age was 63.5 years, and 3598 patients (27.2%) were female individuals. Five of 7 studies^5,9,11,16,19^ included only patients with NSTEACS, while 2 studies (Clopidogrel for the Reduction of Events During Observation [CREDO]^18^ and Antiplatelet Therapy for Reduction of Myocardial Damage During Angioplasty [ARMYDA-5]^17^) included mixed cohorts. Overall, 12 268 patients (92.8%) had a diagnosis of NSTEACS comprised of 7430 (61.7%) NSTEMI and 2109 (31.1%) unstable angina. Access for angiography/PCI was via the radial artery in 4295 (32.6%) and the femoral artery in 8849 (67.3%).

**Table 1.** Clinical Characteristics of Patients Included in the Primary Analysis

Source	Age, mean, y	No. (%)												
		Female patients	Male patients	H	HC	D	Prior MI	Prior PCI	Prior CABG	Presentation			Access	
										NSTEACS	NSTEMI	UA	Radial	Femoral
PCI CURE,^5^ 2001	61.5	804 (30.2)	1854 (69.8)	NA	NA	504 (19.0)	708 (26.6)	361 (13.6)	332 (12.5)	2658 (100)	25.1^a^	74.9^a^	0	2658 (100)
CREDO,^18^ 2002	61.7	606 (28.6)	1510 (71.4)	1450 (68.5)	1580 (74.7)	560 (26.5)	719 (34.0)	NA	NA	1407 (66.5)	290 (13.7)	1117 (52.8)	0	2116 (100)
ARMYDA-5,^17^ 2010	65.5	77 (18.8)	332 (81.2)	305 (74.6)	295 (72.1)	141 (34.5)	137 (33.5)	114 (27.9)	31 (7.6)	160 (39.1)	73 (18.2)	87 (21.3)	40 (9.8)	369 (90.2)
ACCOAST,^9^ 2013	63.7	1110 (27.6)	2923 (72.5)	2504 (62.1)	1814 (45.0)	820 (20.3)	578 (14.3)	661 (16.4)	216 (5.4)	4033 (100)	4033 (100)	0	1711 (42.9)	2276 (57.1)
Bonello et al,^16^ 2015	60.8	54 (25.2)	159 (74.6)	118 (55.3)	105 (49.3)	75 (35.2)	25 (11.5)	26 (12.0)	6 (3.0)	213 (100)	106 (49.8)	107 (50.2)	209 (98.2)	4 (1.9)
ISAR-REACT 5,^11^ 2020	65.8	612 (25.9)	1753 (74.1)	1803 (76.3)	1523 (64.6)	580 (25.8)	443 (18.7)	674 (28.5)	196 (8.3)	2365 (100)	1855 (78.5)	512 (21.5)	983 (41.6)	1359 (57.8)
DUBIUS,^19^ 2020	65	335 (24.5)	1097 (76.6)	942 (67.2)	675 (48.1)	333 (23.8)	245 (17.5)	259 (18.5)	55 (4.0)	1432 (100)	1073 (79.0)	286 (21.0)	1352 (94.5)	67 (5.5)
Overall	63.5	3598 (27.3)	9638 (72.8)	7122 (67.6)	5992 (56.9)	3013 (24.1)	2855 (21.6)	2095 (18.9)	836 (7.5)	12 268 (92.8)	7430 (61.7)	2109 (31.1)	4295 (32.6)	8849 (67.3)

Abbreviations: ACCOAST, A Comparison of Prasugrel at PCI or Time of Diagnosis of Non-ST Elevation Myocardia Infarction; ARMYDA, Antiplatelet Therapy for Reduction of Myocardial Damage During Angioplasty; CABG, coronary artery bypass grafts; CREDO, Clopidogrel for the Reduction of Events During Observation; CURE, Clopidogrel in Unstable Angina to Prevent Recurrent Events Trial; D, diabetes; DUBIUS, Downstream vs Upstream Administration of P2Y12 Receptor Blockers in Non-ST Elevated Acute Coronary Syndromes With Initial Invasive Indication; H, hypertension; HC, hypercholesterolemia; ISAR-REACT, Intracoronary Stenting and Antithrombotic Regimen: Rapid Early Action for Coronary Treatment; MI, myocardial infarction; NA, not applicable; NSTEACS, non-ST elevation acute coronary syndrome; NSTEMI, non-ST elevation myocardial infarction; PCI, percutaneous coronary intervention; UA, unstable angina.

^a^ Absolute number not available.

Key features of each study are presented in Table 2. Four of 7 studies^5,16,17,18^ included only patients undergoing PCI, while 3 studies^9,11,19^ included patients with NSTEACS independent of revascularization strategy. The oral P2Y12 inhibitor used in the pretreatment arm was clopidogrel in 3 studies,^5,17,18^ prasugrel in 1 study,^9^ and ticagrelor in 3 studies.^11,16,19^ Two studies used a different P2Y12 inhibitor in the no pretreatment groups (Intracoronary Stenting and Antithrombotic Regimen: Rapid Early Action for Coronary Treatment [ISAR-REACT 5]^11^ and Bonello et al^16^; both used prasugrel), while 1 study randomized 1:1 within the no pretreatment group between 2 different P2Y12 inhibitors (Downstream vs Upstream Administration of P2Y12 Receptor Blockers in Non-ST Elevated Acute Coronary Syndromes With Initial Invasive Indication [DUBIUS]^19^; 1:1 ticagrelor or prasugrel). All studies mandated dual antiplatelet treatment in both groups until outcome assessment (ie, during the 30-day period after PCI) with the exception of the PCI CURE trial (2220 of 2685 [83.5%] treated with either ticlopidine or clopidogrel; median (IQR) duration, 30 [19-33] days).^5^ Median time from oral P2Y12 inhibitor pretreatment until angiography was within 24 hours for all studies except the PCI CURE study, in which it was 144 hours.^5^ Follow-up outcomes were assessed at 30 days, except for the CREDO trial,^18^ which reported outcomes at 28 days. Two studies^5,9^ reported MACEs occurring between randomization and PCI, with the PCI CURE demonstrating a difference between groups (pretreatment, 47 of 1313 [3.6%]; no pretreatment, 68 of 1345 [5.1%]; *P* = .04), while the A Comparison of Prasugrel at PCI or Time of Diagnosis of Non-ST Elevation MI (ACCOAST) trial found no difference (pretreatment 16 of 2014 [0.8%]; no pretreatment, 18 of 1981 [0.9%]; *P* = .93).

**Table 2.** Key Study Features of Randomized Clinical Trials in the Primary Analysis

Study	Revascularization strategy, No. (%)			Patients, No.			Type and dose of oral P2Y12 Inhibitor		Time to angiography, h	Outcome time point, d
	Medical	PCI	CABG	Overall	Early	Late	Early	Late		
PCI CURE,^5^ 2001	0	2658 (100)	0	2658	1313	1345	Clopidogrel (300 mg LD, 75 mg daily)	Ticlopidine or clopidogrel for 2-4 wk	144	30
CREDO,^18^ 2002	0	2116 (100)	0	2116	1053	1063	Clopidogrel (300 mg LD, 75 mg daily)	Clopidogrel (No LD; 75 mg daily)	6	28
ARMYDA-5,^17^ 2010	0	409 (100)	0	409	204	205	Clopidogrel (600 mg LD, 75 mg daily)	Clopidogrel (600 mg LD, 75 mg daily)	6	30
ACCOAST,^9^ 2013	1014 (25.1)	2770 (68.7)	249 (6.2)	4033	2037	1996	Prasugrel (30 mg LD and 30 mg if PCI with 10 or 5 mg daily)	Prasugrel (60 mg LD, 10 or 5 mg daily)	4.3	30
Bonello et al,^16^ 2015	0	213 (100)	0	213	106	107	Ticagrelor (180 mg LD, 90 mg twice daily)	Prasugrel (60 mg LD, 10 or 5 mg daily)	13.4	30
ISAR-REACT 5,^11^ 2020	477 (20.2)	1809 (76.6)	74 (3.2)	2365	1179	1186	Ticagrelor (180 mg LD, 90 mg twice daily)	Prasugrel (60 mg LD, 10 or 5 mg daily)	1	30
DUBIUS,^19^ 2020	338 (24.1)	970 (69.2)	94 (6.7)	1432	711	721	Ticagrelor (180 mg LD, 90 mg twice daily)	1:1 Randomization to ticagrelor (180 mg LD, 90 mg twice daily) or prasugrel (60 mg LD, 10 or 5mg daily)	23.3	30

Abbreviations: ACCOAST, A Comparison of Prasugrel at PCI or Time of Diagnosis of Non-ST Elevation Myocardia Infarction; ARMYDA, Antiplatelet Therapy for Reduction of Myocardial Damage During Angioplasty; CABG, coronary artery bypass grafts; CREDO, Clopidogrel for the Reduction of Events During Observation; CURE, Clopidogrel in Unstable Angina to Prevent Recurrent Events Trial; DUBIUS, Downstream vs Upstream Administration of P2Y12 Receptor Blockers in Non-ST Elevated Acute Coronary Syndromes With Initial Invasive Indication; ISAR-REACT, Intracoronary Stenting and Antithrombotic Regimen: Rapid Early Action for Coronary Treatment; LD, loading dose; PCI, percutaneous coronary intervention.

Risk-of-bias results found 6 studies^5,9,11,17,18,19^ were at low risk of overall risk of bias, while 1 study^16^ was identified as having moderate risk of bias (eTable 3 and eFigure 1 in the Supplement). Funnel plot distributions and Egger tests for each of the primary, secondary, and primary safety end points demonstrated the absence of publication bias for included studies (eFigure 2A-D in the Supplement), although the funnel plots should be interpreted with some caution given the risk of confounding related to the small number of studies in the meta-analysis.

**Figure 2.**
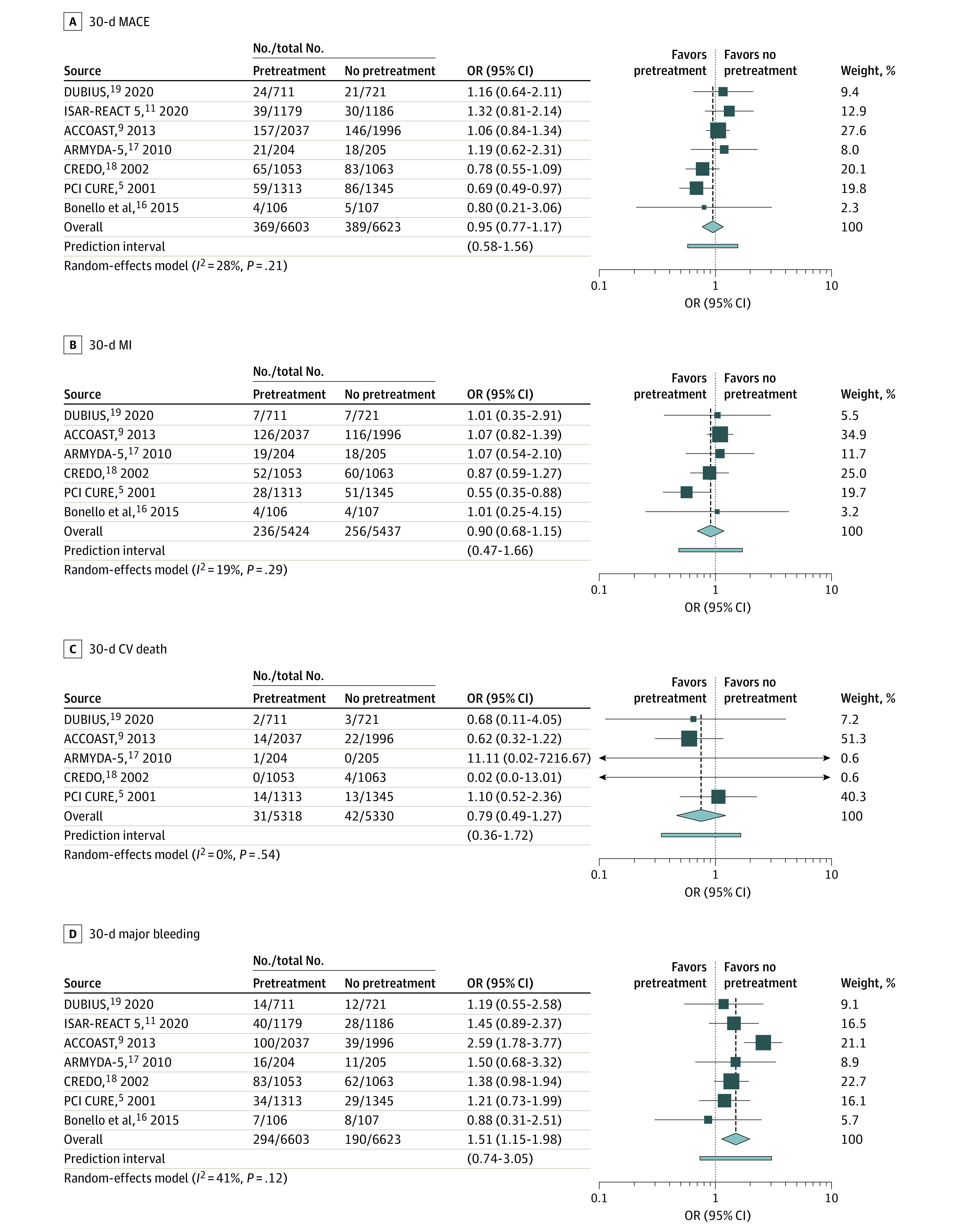
Cardiovascular and Bleeding Outcomes With Oral P2Y12 Inhibitor Pretreatment Compared With No Pretreatment in Patients Non-ST Elevation Acute Coronary Syndromes CV indicates cardiovascular; MACE, major adverse cardiac events; MI, myocardial infarction; and OR, odds ratio.

Cardiovascular and bleeding outcomes are summarized in Figure 2. No differences were observed between patients with oral P2Y12 inhibitor pretreatment and patients without pretreatment for the primary end point of 30-day MACE (OR, 0.95; 95% CI, 0.78-1.15; *I*^2^ = 28%) or secondary end points of 30-day MI (OR, 0.90; 95% CI, 0.72-1.12; *I*^2^ = 19%) and 30-day cardiovascular death (OR, 0.79; 95% CI, 0.49-1.27; *I*^2^ = 0%). Similar results were obtained in a sensitivity analysis using a fixed-effects model (eFigure 3 in the Supplement).

For the primary safety end point, the risk of 30-day major bleeding was higher among patients who underwent pretreatment with an oral P2Y12 inhibitor compared with those who did not (OR, 1.51; 95% CI, 1.16-1.97; *I*^2^ = 41%). The number needed to harm to bring about 1 major bleeding event with oral P2Y12 inhibitor pretreatment was 63 patients.

**Figure 3.**
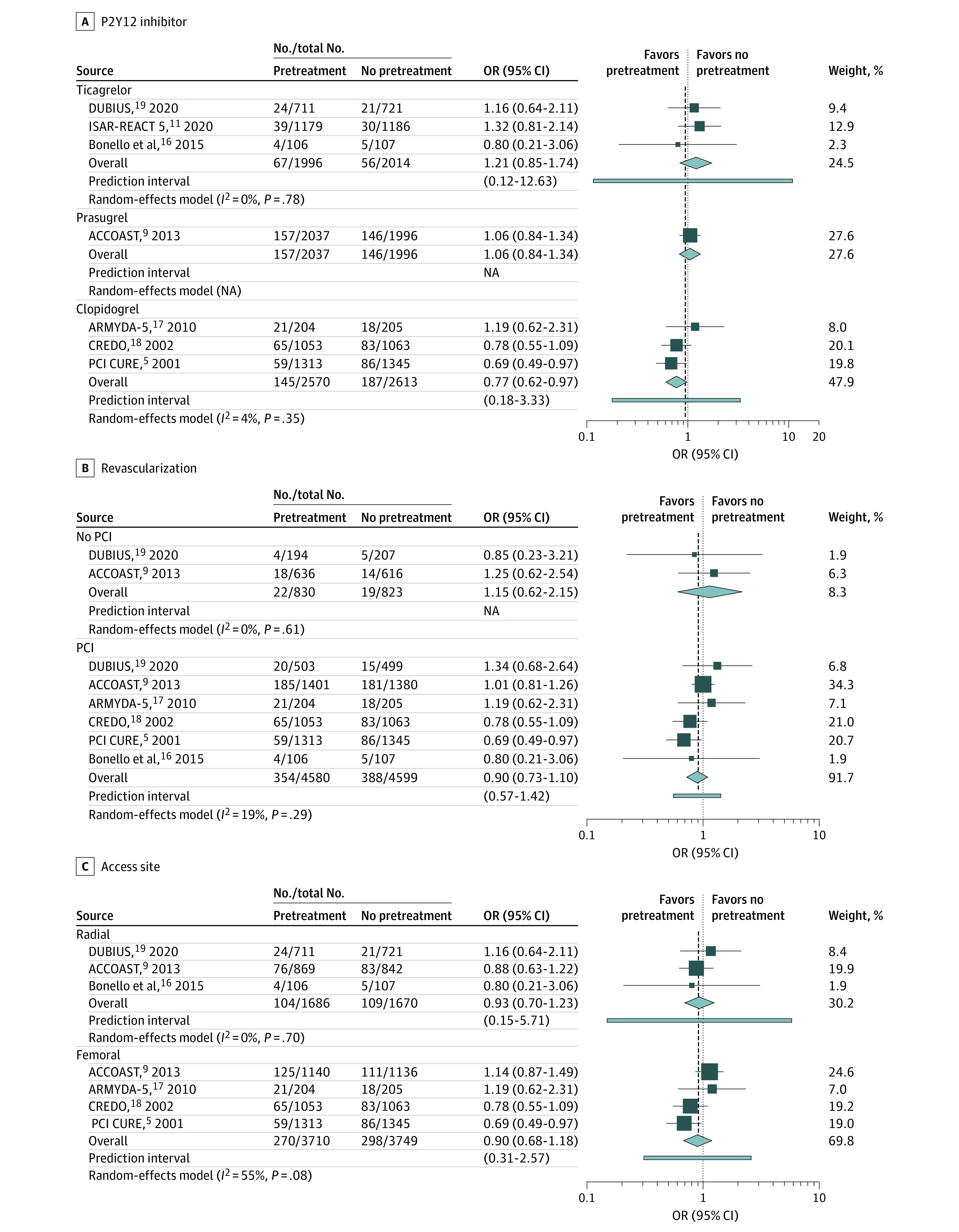
Subgroup Analyses for the Primary End Point of Major Cardiovascular Events Stratified by P2Y12 Inhibitor Used for Pretreatment, Revascularization Strategy, and Arterial Access Site OR indicates odds ratio; and PCI, percutaneous coronary intervention.

In subgroup analysis for the primary end point, the risk of 30-day MACE remained similar between pretreatment and no pretreatment groups when stratified by revascularization strategy (Figure 3B) and arterial access type (Figure 3C). When stratified by oral P2Y12 inhibitor type, risk of 30-day MACE was lower among the pretreatment group receiving clopidogrel pretreatment (OR, 0.77; 95% CI, 0.62-0.97). However, in a post hoc sensitivity analysis excluding the PCI CURE trial, which did not mandate dual antiplatelet treatment during the 30 days after PCI (ie, less reflective of contemporary practice), this finding was no longer present (eFigure 4 in the Supplement).

In subgroup analysis for the primary safety end point, the risk of 30-day major bleeding was no longer significantly increased among patients who underwent radial access (OR, 1.47; 95% CI, 0.77-2.84; *I*^2^ = 41%) or among patients pretreated with ticagrelor (OR, 1.29; 95% CI, 0.88-1.90; *I*^2^ = 0%) (eFigure 5 in the Supplement). Across other subgroups, the risk of 30-day major bleeding was consistent with the primary analysis.

At leave-one-out sensitivity analyses, the results remained consistent with the primary analysis on removal of each study for both the primary and primary safety end points (eFigures 6A-D in the Supplement). We performed a metaregression analysis to assess whether the median time from oral P2Y12 inhibitor pretreatment to angiography at a study level was associated with risk of the primary end point or primary safety end point. For the primary end point, no association was observed between median time between pretreatment and angiography and 30-day MACE. This result was consistent with and without including the PCI CURE trial, which was an outlier because there were a median of 6 days between pretreatment and angiography, compared with less than 24 hours for all other studies (eFigure 2A-B in the Supplement). For the secondary safety end point, no association between timing of pretreatment and risk of 30-day major bleeding was observed (eFigure 7C-D in the Supplement). Finally, to allow comparison, a summary of observational data assessing this topic is presented in eTable 4 in the Supplement.

## Discussion

In this meta-analysis of 7 RCTs enrolling 13 226 patients, the major findings are as follows: (1) pretreatment with oral P2Y12 inhibitors prior to angiography was not associated with a lower risk of short-term MACEs, MI, or cardiovascular death among patients with NSTEACS; (2) the risk of bleeding was increased among patients receiving pretreatment with oral P2Y12 inhibitors; (3) findings were generally consistent when stratified by oral P2Y12 inhibitor type, revascularization strategy, and arterial access site, with the exception of reduced MACEs among the group receiving clopidogrel treatment (reliant on the PCI CURE trial) and no significant difference in bleeding risk among patients undergoing surgery with radial access.

Pretreatment with oral P2Y12 inhibitors in patients with NSTEACS has been extensively discussed over the last 2 decades.^2,3^ Such a strategy has some clinical rationale, with the goal of achieving greater platelet inhibition at the time of PCI and potentially offering a protective effect against further ischemic events while awaiting angiography. However, randomized data do not support this approach. Until last year, European guidelines have recommended pretreatment with oral P2Y12 inhibitors in patients with NSTEACS with the caveat of limiting this advice to ticagrelor and clopidogrel, given the results of the ACCOAST trial, which showed no benefit with prasugrel pretreatment.^7,8,9^ US guidelines have remained more cautious but are yet to recommend against the use of routine pretreatment. European recommendations had been largely based on the PCI CURE and CREDO trials, which showed potential benefits of early clopidogrel, and subsequently the PLATO trial, which demonstrated the superiority of ticagrelor pretreatment vs clopidogrel pretreatment.^5,6,18^ These studies had several limitations with regards to drawing conclusions surrounding the efficacy of pretreatment. In the PCI CURE trial (included in this study), median time from clopidogrel pretreatment was 6 days, which has limited applicability to contemporary recommendations for early invasive strategy (ie, <24 hours) in most patients with NSTEACS.^5,8^ Furthermore, patients were treated with either clopidogrel or ticlopidine for 2 to 4 weeks after PCI according to local practices at the time, which is inconsistent with the uninterrupted dual antiplatelet practices of the last 15 years. In the CREDO trial, differences in early ischemic outcomes were limited to the subgroup of patients receiving clopidogrel more than 6 hours before PCI, and of note, the no pretreatment group started on 75 mg daily clopidogrel following PCI without a loading dose.^18^ Moreover, the CREDO trial was negative for its primary end point, which limits interpretation of any subgroup analyses. Finally, in the PLATO trial, both ticagrelor and clopidogrel were given using a pretreatment strategy, making it difficult to draw any conclusions regarding the use of pretreatment for ticagrelor.^6^ Nonetheless, these studies formed the basis for the recommendation for routine pretreatment in NSTEACS until it was changed last year based on the ACCOAST trial, ISAR-REACT 5 trial, and observational studies.^8,9,10,11^

Our study has several advantages compared with the data available at the time of the 2020 European Society of Cardiology NSTEACS guidelines and the 2014 meta-analysis.^1,8^ Compared with the previous meta-analysis, we include 4 additional randomized trials. The resultant increase in sample size meant that we were able to exclude observational studies, thus avoiding potential bias associated with their inclusion. Results appeared consistent across both NSTEACS and NSTEMI, with the ACCOAST trial including exclusively patients who were troponin positive and the ARMYDA-5 study presenting ACS subtype data.^9,17^ Recent data included in this meta-analysis have used the third universal definition for MI, requiring an increase and decrease of troponin levels in addition to symptoms, electrocardiogram changes, or new angiographic or imaging abnormalities.^11,19,20^ Bonello et al^16^ found an association of ticagrelor pretreatment with reduced rates of periprocedural myonecrosis (5 times >99th percentile and >20% increase) and myocardial injury (>99th percentile); however, this observation is within the limitations of a small trial, and the clinical relevance of these measures remains debated.^21^ In the significantly larger ALPHEUS trial, aggressive P2Y12 inhibition with ticagrelor did not reduce periprocedural myocardial necrosis in elective PCI.^22^ Data regarding pretreatment with each type of oral P2Y12 inhibitor (ie, clopidogrel, ticagrelor, and prasugrel) were included in our analysis, providing evidence for a class effect rather than an isolated finding for prasugrel alone. Evidence regarding ticagrelor has been needed in particular, and the 3 RCTs assessing ticagrelor pretreatment consistently showed no benefit on ischemic end points. Although the no pretreatment groups used prasugrel in 2 of these trials,^11,16^ these 2 studies, in combination with the DUBIUS trial,^19^ should be sufficient to reasonably conclude a lack of benefit with ticagrelor pretreatment. Recent observational data support this conclusion, with data from the Swedish Coronary Angiography and Angioplasty Registry (64 857 participants) demonstrating no difference in ischemic end points and increased bleeding risk among patients pretreated with oral P2Y12 inhibitors (34 785 [54.5%] received ticagrelor; 27 867 [43.7%], clopidogrel; 1148 [1.8%], prasugrel), which is supported by data from smaller registries.^10,23,24^

While these results suggest that routine P2Y12 inhibitor pretreatment should be avoided for most patients with NSTEACS, a caveat remains for patients who are selected for a conservative treatment strategy or for whom a prolonged delay to angiography (ie, >72 hours) is necessitated (eg, transfer from a non-PCI center). Outside of the PCI CURE trial, all trials in this study had a median time to angiography within 24 hours, which in some circumstances may not be representative of real-world practice. A recent analysis of patients undergoing inpatient angiography for NSTEACS from the National Cardiovascular Data Registry Acute Coronary Treatment and Intervention Outcomes Network registry found that angiography occurred later than 24 hours and later than 72 hours in 42% and 8% of patients, respectively.^25^ The DUBIUS trial^19^ is likely the most reflective of this scenario, and in this trial, 51% of patients underwent angiography later than 24 hours after presentation. No differences in ischemic end points were observed in subgroup analysis limited to this group, but it is difficult to generalize this finding to delays greater than 72 hours. In the ACCOAST substudy addressing interval to angiography,^26^ no differences in ischemic end points were observed across quartiles of increasing pretreatment duration, although the greatest duration quartile remained within 24 hours (ie, >13.6 hours). The PCI CURE trial is the only study to assess a prolonged delay to angiography (median, 6 days), and the results of the clopidogrel subgroup analysis suggest pretreatment may confer a potential benefit in patients with prolonged delays to angiography. Nonetheless, selection of these patients should be done carefully given the potential for harm. Similarly, given that an early invasive strategy is the goal for most patients, it is likely to be difficult to identify which patients will encounter prolonged delays before the delay has occurred.

In our analysis, two-thirds of patients underwent femoral access, which is different from the contemporary practice of radial access. Our subgroup analysis limited to radial access demonstrated no increase in bleeding risk with pretreatment, although with some limitations of this result. ISAR-REACT 5 did not present data regarding early bleeding risk separated by access site, and the ACCOAST trial presented only 7-day Thrombolysis in MI (TIMI) major bleeding in subgroup data (whereas TIMI major and minor is more reflective of Bleeding Academic Research Consortium [BARC] score ≥3). Therefore, our subgroup analysis for bleeding risk with radial access was limited to 3 trials and, with an altered definition for 1 trial, resulted in a lower event rate than the primary analysis. Multiple studies have demonstrated substantially lower bleeding rates in radial compared with femoral access,^27^ and therefore the number needed to harm estimated in this study (ie, 63 patients) may overestimate the bleeding risk of pretreatment among contemporary cohorts undergoing radial access. Conversely, the ACCOAST trial demonstrated no interaction between access site and the observed increase in bleeding risk.^28^ Nevertheless, decisions regarding access site may not be known at the time of pretreatment; multiple recent observational studies have demonstrated pretreatment is associated with increased bleeding risk among patients who require bypass surgery or among those with an alternate diagnosis (eg, aortic dissection)^29,30,31^; and most importantly, no benefit in regards to ischemic end points was observed.

### Limitations

There are a number of limitations to this meta-analysis. First, as with any meta-analysis, the results are dependent on the availability and quality of reported data. Two studies^17,18^ included a proportion of patients without NSTEACS, which were included in this analysis because outcomes were not presented for the NSTEACS groups alone. Similarly, several studies did not present cardiovascular or bleeding outcomes for each subgroup at the time of interest. For example, while 3 studies^9,11,19^ included patients who were not managed with PCI, only 1 study reported bleeding outcomes for the subgroup not undergoing PCI and this was limited to 7-day rather than 30-day outcomes.^9^ In this setting, the sample size for some subgroups was small, limiting their power. Second, different bleeding definitions were used, with more recent studies using BARC criteria and older studies using TIMI criteria, limiting the reliability of the pooled estimate for the secondary safety end point. Minor differences in ischemic end points were also present. Third, 2 studies used different P2Y12 inhibitors between the pretreatment and no pretreatment groups, which may have introduced bias into the analyses. However, we attempted to account for this using the leave-one-out sensitivity analysis, which showed results remained consistent on exclusion of these 2 trials. Fourth, time between pretreatment and angiography varied between studies, and while we aimed to assess this using the metaregression analysis, this should only be considered hypothesis generating given the low number of studies included in the analysis.

## Conclusions

In this systematic review and meta-analysis, pretreatment with oral P2Y12 inhibitors prior to angiography, compared with treatment at the time of PCI once coronary anatomy is known, was associated with increased bleeding risk and no difference in cardiovascular outcomes. A strategy of routine oral P2Y12 inhibitor administration at time of NSTEACS diagnosis among patients planned for angiography was not supported by this study and may be harmful.

## Notes

Supplement. **eTable 1. **Details of Search Strategy **eTable 2. **End Point Definitions by Study **eTable 3. **Risk of Bias (RoB 2) Output **eTable 4. **Summary of Observational Studies Available Assessing P2Y12 Inhibitor Timing in NSTEACS **eFigure 1. **Risk of Bias (RoB 2) Summary **eFigure 2. **Funnel Plots **eFigure 3. **Sensitivity Analysis Using a Fixed-Effects Model **eFigure 4. **Sensitivity Analysis Excluding PCI CURE From P2Y12 Inhibitor Subgroup Analysis **eFigure 5. **Subgroup Analyses for the Primary Safety End Point (Bleeding) **eFigure 6. **Leave-One-Out Sensitivity Analyses **eFigure 7. **Metaregression Assessing Timing of Pretreatment Loading Dose
eReferences.
