# Supplementary material for: Assessment of Pretreatment With Oral P2Y12 Inhibitors and Cardiovascular and Bleeding Outcomes in Patients With Non-ST Elevation Acute Coronary Syndromes: A Systematic Review and Meta-analysis
Source: JAMA Netw Open. 2021 Nov 19;4(11):e2134322. doi: 10.1001/jamanetworkopen.2021.34322 (PMC8605486; doi:10.1001/jamanetworkopen.2021.34322)
Supplement: Supplement. — eTable 1. Details of Search Strategy eTable 2. End Point Definitions by Study eTable 3. Risk of Bias (RoB 2) Output eTable 4. Summary of Observational Studies Available Assessing P2Y12 Inhibitor Timing in NSTEACS eFigure 1. Risk of Bias (RoB 2) Summary eFigure 2. Funnel Plots eFigure 3. Sensitivity Analysis Using a Fixed-Effects Model eFigure 4. Sensitivity Analysis Excluding PCI CURE From P2Y12 Inhibitor Subgroup Analysis eFigure 5. Subgroup Analyses for the Primary Safety End Point (Bleeding) eFigure 6. Leave-One-Out Sensitivity Analyses eFigure 7. Metaregression Assessing Timing of Pretreatment Loading Dose eReferences. [file jamanetwopen-e2134322-s001.pdf]

Supplemental Online Content

Dawson LP, Chen D, Dagan M, et al. Assessment of pretreatment with oral P2Y12 inhibitors and cardiovascular and bleeding outcomes in patients with non-ST elevation acute coronary syndromes. *JAMA Netw Open.* 2021;4(11):e2134322. doi:10.1001/jamanetworkopen.2021.34322

eTable 1. Details of Search Strategy

eTable 2. End Point Definitions by Study

eTable 3. Risk of Bias (RoB 2) Output

eTable 4. Summary of Observational Studies Available Assessing P2Y12 Inhibitor Timing in NSTEACS

eFigure 1. Risk of Bias (RoB 2) Summary

eFigure 2. Funnel Plots

eFigure 3. Sensitivity Analysis Using a Fixed-Effects Model

eFigure 4. Sensitivity Analysis Excluding PCI CURE From P2Y12 Inhibitor Subgroup Analysis

eFigure 5. Subgroup Analyses for the Primary Safety End Point (Bleeding)

eFigure 6. Leave-One-Out Sensitivity Analyses

eFigure 7. Metaregression Assessing Timing of Pretreatment Loading Dose

eReferences.

This supplemental material has been provided by the authors to give readers additional information about their work.

© 2021 Dawson LP et al. *JAMA Network Open.*

eTable 1. Details of Search Strategy

Database	Search Strategy	Results
MEDLINE	[pretreatment.mp OR upstream.mp OR up front.mp OR before PCI.mp OR early.mp OR loading.mp OR timing.mp OR pre-hospital.mp] AND [P2Y12 inhibitor.mp OR Purinergic P2Y Receptor Antagonist/ OR clopidogrel.mp OR Clopidogrel/ OR prasugrel.mp OR Prasugrel Hydrochloride/ OR ticagrelor.mp OR Ticagrelor/] AND [Percutaneous Coronary Intervention/ OR Acute Coronary Syndrome/ OR Non-ST Elevated Myocardial Infarction/ OR nsteacs.mp OR Coronary Angiography/ or Myocardial Infarction/ OR ischemic heart disease.mp OR Myocardial Ischemia/]; limited to humans, English, and year 2000 to current	1336
PubMed	(("P2Y12"[All Fields] AND ("antagonists and inhibitors"[MeSH Subheading] OR ("antagonists"[All Fields] AND "inhibitors"[All Fields]) OR "antagonists and inhibitors"[All Fields] OR "inhibitors"[All Fields] OR "inhibitor"[All Fields] OR "inhibitor s"[All Fields])) OR ("clopidogrel"[MeSH Terms] OR "clopidogrel"[All Fields] OR "clopidogrel s"[All Fields]) OR ("prasugrel hydrochloride"[MeSH Terms] OR ("prasugrel"[All Fields] AND "hydrochloride"[All Fields]) OR "prasugrel hydrochloride"[All Fields] OR "prasugrel"[All Fields] OR "prasugrel s"[All Fields]) OR ("ticagrelor"[MeSH Terms] OR "ticagrelor"[All Fields])) AND ("non st elevated myocardial infarction"[MeSH Terms] OR ("non-st"[All Fields] AND "elevated"[All Fields] AND "myocardial"[All Fields] AND "infarction"[All Fields]) OR "non st elevated myocardial infarction"[All Fields] OR ("non"[All Fields] AND "st"[All Fields] AND "elevation"[All Fields] AND "myocardial"[All Fields] AND "infarction"[All Fields]) OR "non st elevation myocardial infarction"[All Fields] OR ("non-st"[All Fields] AND ("elevate"[All Fields] OR "elevated"[All Fields] OR "elevates"[All Fields] OR "elevating"[All Fields] OR "elevation"[All Fields] OR "elevational"[All Fields] OR "elevations"[All Fields]) AND ("acute coronary syndrome"[MeSH Terms] OR ("acute"[All Fields] AND "coronary"[All Fields] AND "syndrome"[All Fields]) OR "acute coronary syndrome"[All Fields])) OR ("acute coronary syndrome"[MeSH Terms] OR ("acute"[All Fields] AND "coronary"[All Fields] AND "syndrome"[All Fields]) OR "acute coronary syndrome"[All Fields])) AND ("pretreat"[All Fields] OR "pretreated"[All Fields] OR "pretreating"[All Fields] OR "pretreatment"[All Fields] OR "pretreatments"[All Fields] OR ("upstream"[All Fields] OR "upstreams"[All Fields]) OR ("up"[All Fields] AND ("front"[All Fields] OR "front s"[All Fields] OR "fronts"[All Fields])) OR "early"[All Fields] OR ("loaded"[All Fields] OR "loading"[All Fields] OR "loadings"[All Fields] OR "loads"[All Fields]) OR ("timely"[All Fields] OR "timing"[All Fields] OR "timings"[All Fields]) OR "pre-hospital"[All Fields])	1064
Embase	[pretreatment.mp OR upstream.mp OR up front.mp OR before PCI.mp OR early.mp OR loading.mp OR timing.mp OR pre-hospital.mp] AND [clopidogrel/ OR purinergic P2Y12 receptor/ OR prasugrel/ OR P2Y12 inhibitor.mp OR ticagrelor/] AND [acute coronary syndrome.mp OR acute coronary syndrome/ OR non-st elevation myocardial infarction.mp OR non ST segment elevation myocardial infarction/ or non st segment elevation acute coronary syndrome/ or nsteacs.mp]; limited to humans and English language and yr="2000-Current"	4166
Scopus	(pretreatment OR upstream OR "up front" OR "before PCI" OR early OR loading OR timing OR pre-hospital) AND ("P2Y12 inhibitor" OR clopidogrel OR prasugrel OR ticagrelor) AND ("non-ST elevation myocardial infarction" OR "non-ST acute coronary syndrome") AND (LIMIT-TO (PUBYEAR, 2021) OR LIMIT-TO (PUBYEAR, 2020) OR LIMIT-TO (PUBYEAR, 2019) OR LIMIT-TO (PUBYEAR, 2018) OR LIMIT-TO (PUBYEAR, 2017) OR LIMIT-TO (PUBYEAR, 2016) OR LIMIT-TO (PUBYEAR, 2015) OR LIMIT-TO (PUBYEAR, 2014) OR LIMIT-TO (PUBYEAR, 2013) OR LIMIT-TO (PUBYEAR, 2012) OR LIMIT-TO (PUBYEAR, 2011) OR LIMIT-TO (PUBYEAR, 2010) OR LIMIT-TO (PUBYEAR, 2009) OR LIMIT-TO (PUBYEAR, 2008) OR LIMIT-TO (PUBYEAR, 2007) OR LIMIT-TO (PUBYEAR, 2006) OR LIMIT-TO (PUBYEAR, 2005) OR LIMIT-TO (PUBYEAR, 2004) OR LIMIT-TO (PUBYEAR, 2003) OR LIMIT-TO (PUBYEAR, 2002) OR LIMIT-TO (PUBYEAR, 2001) OR LIMIT-TO (PUBYEAR, 2000)) AND (LIMIT-TO (LANGUAGE, "English"))	3532
Web of Science	ALL=(pretreatment OR upstream OR "up front" OR "before PCI" OR early OR loading OR timing OR pre-hospital) AND ALL=("P2Y12 inhibitor" OR clopidogrel OR ticagrelor OR prasugrel) AND ALL=("acute coronary syndrome" OR "non-ST elevation myocardial infarction" OR "non-ST acute coronary syndrome") AND LANGUAGE: (English) Indexes=SCI-EXPANDED, SSCI, A&HCI, CPCI-S, CPCI-SSH, BKCI-S, BKCI-SSH, ESCI, CCR-EXPANDED, IC Timespan=2000-2021	1225
Science Direct	(pretreatment OR timing OR early OR upstream) AND (clopidogrel OR ticagrelor OR prasugrel) AND ("non-ST elevation myocardial infarction" OR "non-ST acute coronary syndrome"); Year(s) 2000-2021	1360
clinicaltrials.gov	(pretreatment OR upstream OR up front OR before PCI OR early OR loading OR timing OR pre-hospital) AND (P2Y12 inhibitor OR clopidogrel OR ticagrelor OR prasugrel) AND (acute coronary syndrome OR non-ST elevation myocardial infarction)	154
Cochrane Central Register	[(coronary angiography) OR (myocardial infarction) OR ("ischaemic heart disease") OR (ischemic heart disease) OR ("myocardial ischemia") OR (percutaneous coronary intervention) OR ("percutaneous coronary revascularisation") OR (acute coronary syndrome) OR ("NSTEMI")	1819

© 2021 Dawson LP et al. *JAMA Network Open.*

of Controlled Trials	OR (nsteacs)] AND [("loading dose") OR (loading) OR (timing) OR ("pre-hospital treatment") OR (pre-hospital) OR (pretreatment) OR (upstream) OR (up front) OR (before PCI) OR (early)] AND [(prasugrel) OR (clopidogrel) OR (ticagrelor) OR (P2Y12 inhibitor); with Publication Year from 2000 to 2021, in Trials (Word variations have been searched)	

© 2021 Dawson LP et al. *JAMA Network Open.*

eTable 2. End Point Definitions by Study

Study	Time point	Primary endpoint (MACE)		Major Bleeding
CURE PCI[1]	30d	CV death, MI, urgent target vessel revascularisation		Major (fatal, Hb drop ≥50g/L, caused hypotension requiring inotropes or surgery, intracranial haemorrhage, ≥4 units transfusion) or minor (interruption of study medication)
CREDO[2]	28d	Death, MI, urgent target vessel revascularisation		TIMI major or minor
ARMYDA-5[3]	30d	CV death, MI, target vessel revascularisation		TIMI major or minor
ACCOAST[4, 5]	30d	CV death, MI, urgent revascularisation		TIMI major or minor
Bonello et al[6]	30d	CV death, MI, urgent revascularisation, stroke		BARC score 3,4,5
ISAR-REACT 5[7]	30d	Death, MI, stroke		BARC score 3,4,5
DUBIUS[8]	30d	CV death, MI, non-fatal stroke, major or fatal bleeding (BARC>2)		BARC score 3,4,5

TIMI major: intracranial haemorrhage, ≥5g/dl decrease in Hb, or ≥15% decrease in Hct
TIMI minor: 3-5g/dl decrease in Hb with clinically overt bleeding
BARC 3: overt bleeding plus Hb drop of ≥3/dL, any transfusion, intracranial haemorrhage, tamponade, bleeding requiring surgical intervention
BARC 4: CABG related bleeding, perioperative intracranial bleeding within 48h, reoperation after closure of sternotomy to control bleeding, transfusion ≥5 units within 48h period, chest tube output ≥2L within 24 hours
BARC 5: fatal bleeding
Mehran 2011

© 2021 Dawson LP et al. *JAMA Network Open.*

eTable 3. Risk of Bias (RoB 2) Output

Question	DUBIUS	ISAR-REACT5	ACCOAST	ARMYDA-5	CREDO	Bonello et al	CURE PCI
1.1	Yes	Yes	Yes	Yes	Yes	Yes	Yes
1.2	Yes	Yes	Yes	Probably yes	Yes	Probably yes	Yes
1.3	No	No	No	No	No	No	No
**1 Overall**	**Low risk**	**Low risk**	**Low risk**	**Low risk**	**Low risk**	**Low risk**	**Low risk**
2.1	Yes	Yes	No	Yes	No	Yes	No
2.2	Yes	Yes	No	Yes	No	Yes	No
2.3	No	No	n/a	No	n/a	No	n/a
2.4	n/a	n/a	n/a	n/a	n/a	n/a	n/a
2.5	n/a	n/a	n/a	n/a	n/a	n/a	n/a
2.6	Yes	Yes	Yes	Yes	Yes	No information	Yes
2.7	n/a	n/a	n/a	n/a	n/a	n/a	n/a
**2 Overall**	**Low risk**	**Low risk**	**Low risk**	**Low risk**	**Low risk**	**Some concerns**	**Low risk**
3.1	Yes	Yes	Yes	Probably yes	Yes	Yes	Yes
3.2	n/a	n/a	n/a	n/a	n/a	n/a	n/a
3.3	n/a	n/a	n/a	n/a	n/a	n/a	n/a
3.4	n/a	n/a	n/a	n/a	n/a	n/a	n/a
**3 Overall**	**Low risk**	**Low risk**	**Low risk**	**Low risk**	**Low risk**	**Low risk**	**Low risk**
4.1	No	No	No	No	No	No	No
4.2	No	No	No	No	No	No	No
4.3	n/a	n/a	n/a	Yes	n/a	n/a	n/a
4.4	n/a	n/a	n/a	No	n/a	n/a	n/a
4.5	n/a	n/a	n/a	n/a	n/a	n/a	n/a
**4 Overall**	**Low risk**	**Low risk**	**Low risk**	**Low risk**	**Low risk**	**Low risk**	**Low risk**
5.1	Yes	Yes	Yes	Yes	Yes	No information	Yes
5.2	No	No	No	No	No	Probably no	No
5.3	No	No	No	No	No	Probably no	No
**5 Overall**	**Low risk**	**Low risk**	**Low risk**	**Low risk**	**Low risk**	**Some concerns**	**Low risk**
**Overall RoB**	**Low risk**	**Low risk**	**Low risk**	**Low risk**	**Low risk**	**Some concerns**	**Low risk**

eTable 4. Summary of Observational Studies Available Assessing P2Y12 Inhibitor Timing in NSTEACS

Study	Year	N	Population	Pre-treatment	Impact on MACE	Impact on bleeding	Results
Dworeck et al[9]	2020	64,857	NSTEACS	Any	-	↑	Pre-treatment not associated with improved clinical outcomes but was associated with increased risk of bleeding.
Verdoia et al[10]	2018	168	SIHD/ NSTEACS	Any	-	n/a	No difference in MI or periprocedural myonecrosis.
Sukul et al[11]	2017	24,733	Mixed	Any	-	-	No differences in outcomes between pre-treatment and no pre-treatment.
Yudi et al[12]	2015	6,817	NSTEACS	Any	-	-	No difference in 30-day mortality, MACE or bleeding complications.
Ikegami et al[13]	2015	6,528	ACS	Any	↓	-	Pre-treatment associated with reduced MACE and no difference in bleeding endpoints.
Almendro-delia et al[14]	2015	9,621	NSTEACS	Clopidogrel	-	↑	No difference in reinfarction, stent thrombosis, or mortality among NSTEACS subgroup.
Wang et al[15]	2011	9,166	NSTEACS	Clopidogrel	-	↑	No interaction between upstream clopidogrel administration with randomized eptifibatide for MACE, but increased risk of bleeding with both (EARLY ACS substudy).
Feldman et al[16]	2010	1,041	NSTEACS	Clopidogrel	-	-	No difference in MACE or bleeding complications.
Lincoff et al[17]	2008	13,819	NSTEACS	Clopidogrel	↓	-	Post-hoc analysis of the ACUITY trial, increase in ischemic events for patients not pre-treated if randomized to bivalirudin
Szuk et al[18]	2007	4,160	Mixed	Clopidogrel	↓	↑	Pre-treatment associated with increased MACE and higher major bleeding.
Dery et al[19]	2007	2,040	Mixed	Clopidogrel	↓	-	Pre-treatment associated with reduced MACE and no impact on bleeding (ESPRIT substudy.).
Chan et al[20]	2003	4,809	Mixed	Clopidogrel	↓	-	Clopidogrel pre-treatment associated with reduced death and MI irrespective of GP IIb/IIIa inhibitor used (TARGET substudy).
Assali et al[21]	2001	299	Mixed	Clopidogrel	↓	-	Reduced MACE and no difference on bleeding endpoints

eFigure 1. Risk of Bias (RoB 2) Summary
Upper panel shows weighted summary for all studies, while lower panel shows risk of bias by domain for each individual study.

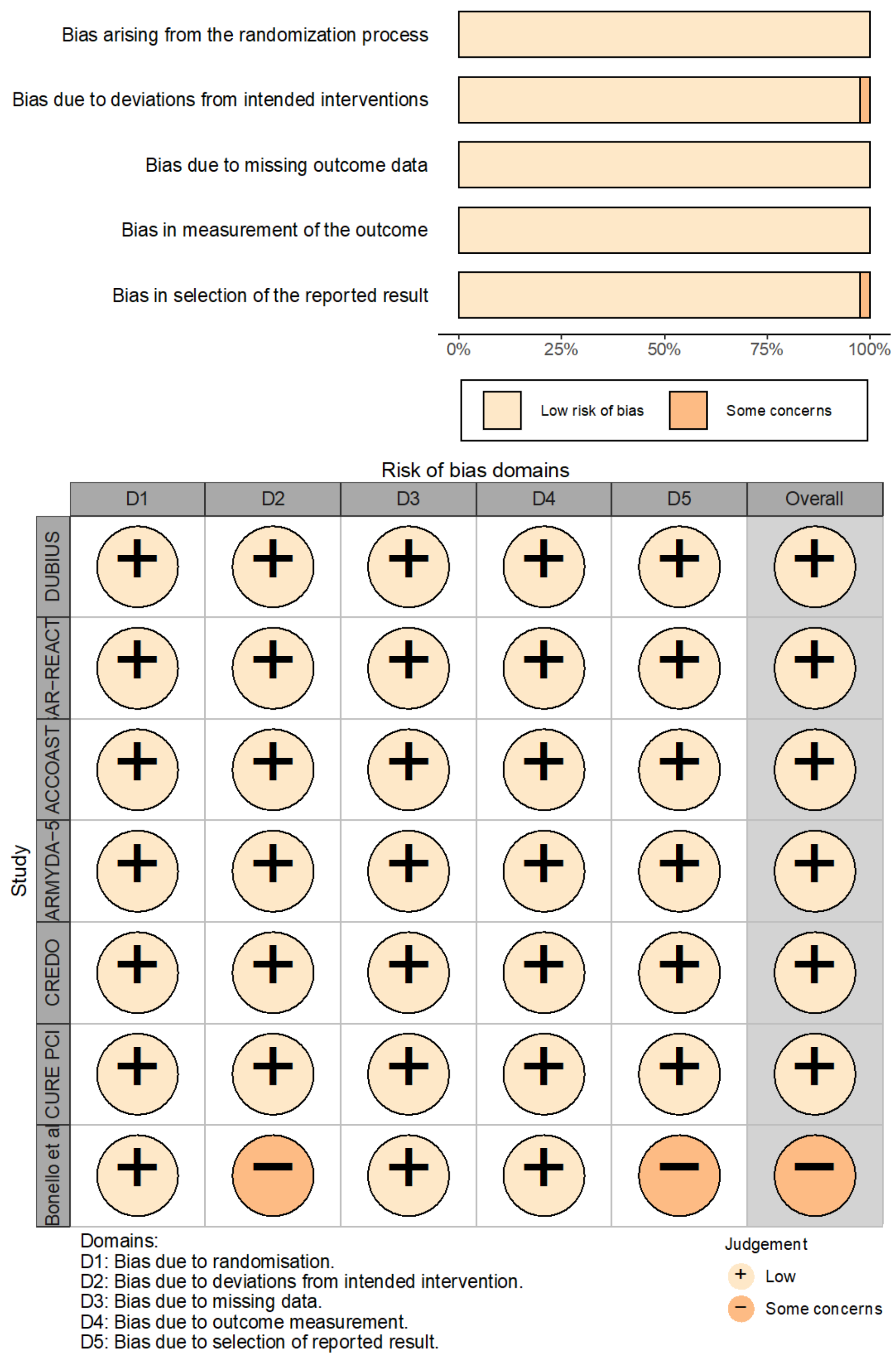

© 2021 Dawson LP et al. *JAMA Network Open.*

eFigure 2. Funnel Plots

eFigure 2a. Funnel plot for 30-day major adverse cardiac events (Egger's test, p=0.78)

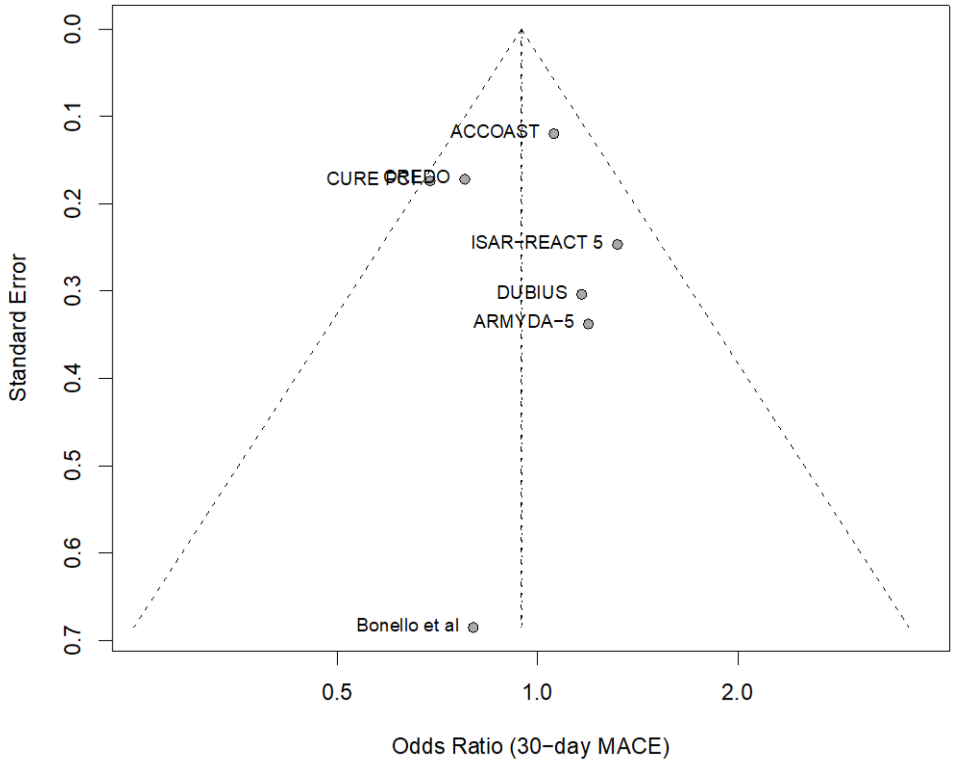

eFigure 2b. Funnel plot for 30-day myocardial infarction (Egger's test, p=0.73)

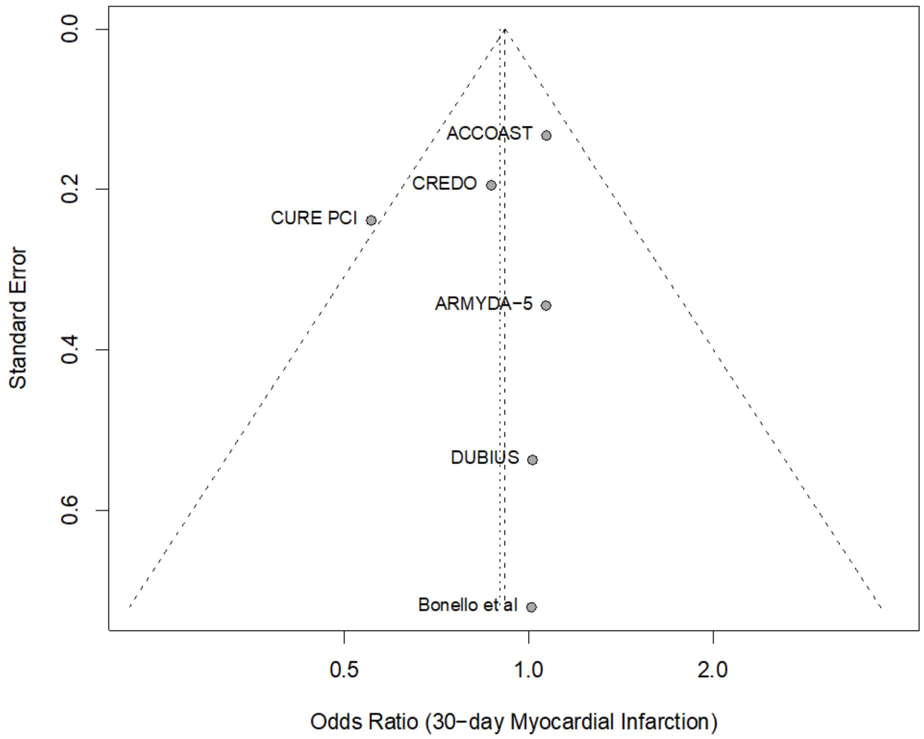

© 2021 Dawson LP et al. *JAMA Network Open.*

eFigure 2c. Funnel plot for 30-day cardiovascular death (Egger's test, p=0.87)

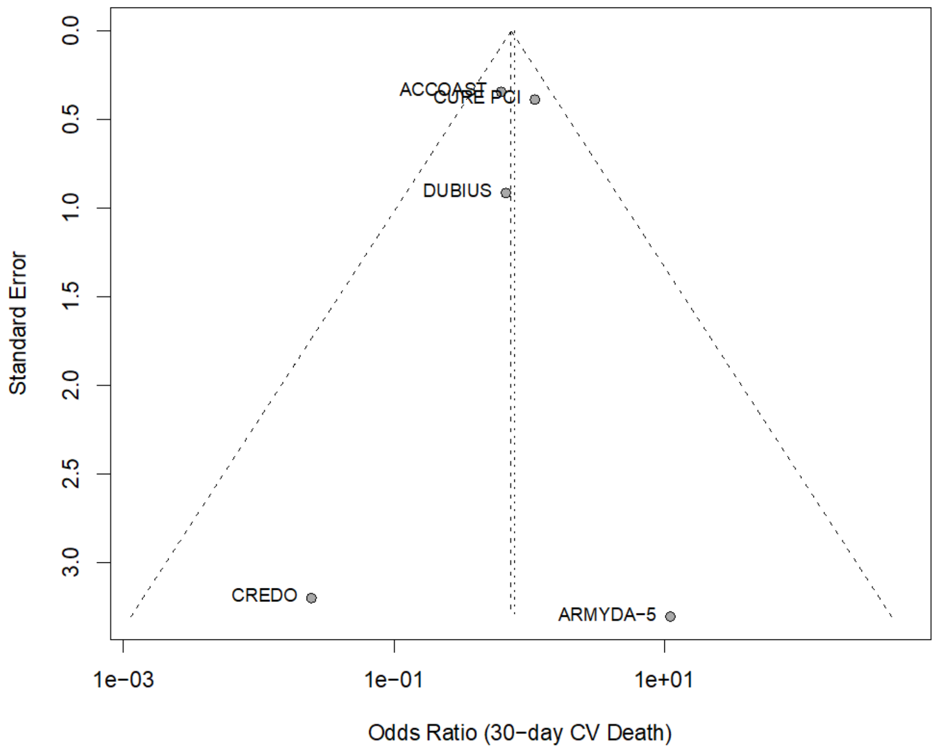

eFigure 2d. Funnel plot for 30-day major bleeding (Egger's test, p=0.29)

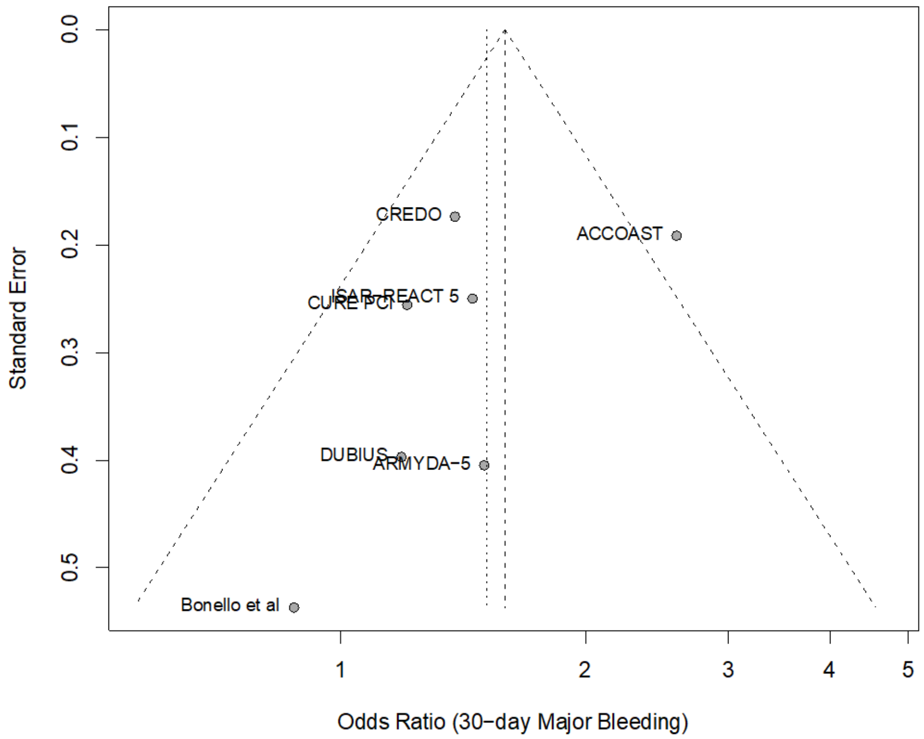

© 2021 Dawson LP et al. *JAMA Network Open.*

eFigure 3. Sensitivity Analysis Using a Fixed-Effects Model

(A) 30-day MACE, (B) 30-day myocardial infarction, (C) 30-day cardiovascular death, and (D) 30-day major bleeding.

A

30–day MACE	Early n	Early N	Late n	Late N	OR	95% CI	Weight
DUBIUS	24	711	21	721	1.16	[0.64; 2.11]	5.5%
ISAR–REACT 5	39	1179	30	1186	1.32	[0.81; 2.14]	7.9%
ACCOAST	157	2037	146	1996	1.06	[0.84; 1.34]	37.3%
ARMYDA–5	21	204	18	205	1.19	[0.62; 2.31]	4.4%
CREDO	65	1053	83	1063	0.78	[0.55; 1.09]	21.3%
CURE PCI	59	1313	86	1345	0.69	[0.49; 0.97]	22.2%
Bonello et al	4	106	5	107	0.80	[0.21; 3.06]	1.3%
**Fixed effect model**		**6603**		**6623**	**0.95**	**[0.82; 1.10]**	**100.0%**

Heterogeneity: $I^2 = 28\%$, $p = 0.21$

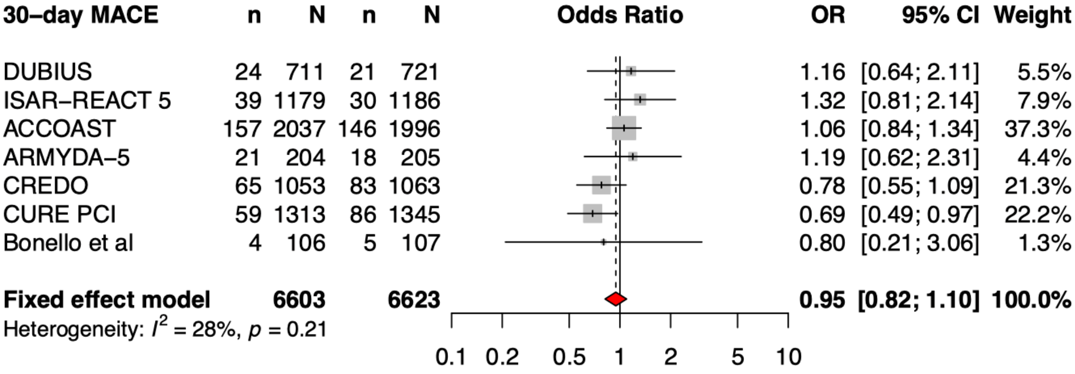

B

30–day MI	Early n	Early N	Late n	Late N	OR	95% CI	Weight
DUBIUS	7	711	7	721	1.01	[0.35; 2.91]	2.8%
ACCOAST	126	2037	116	1996	1.07	[0.82; 1.39]	45.2%
ARMYDA–5	19	204	18	205	1.07	[0.54; 2.10]	6.7%
CREDO	52	1053	60	1063	0.87	[0.59; 1.27]	23.4%
CURE PCI	28	1313	51	1345	0.55	[0.35; 0.88]	20.3%
Bonello et al	4	106	4	107	1.01	[0.25; 4.15]	1.6%
**Fixed effect model**		**5424**		**5437**	**0.91**	**[0.76; 1.10]**	**100.0%**

Heterogeneity: $I^2 = 19\%$, $p = 0.29$

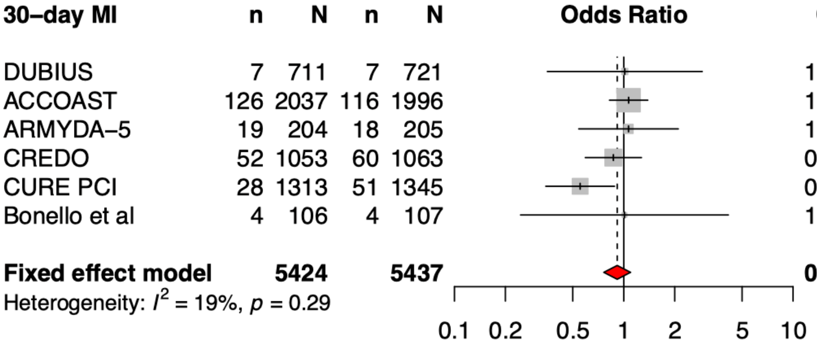

© 2021 Dawson LP et al. *JAMA Network Open.*

C

30-day CV Death	Early n	Early N	Late n	Late N	Odds Ratio	OR	95% CI	Weight
DUBIUS	2	711	3	721		0.68	[0.11; 4.05]	7.1%
ACCOAST	14	2037	22	1996		0.62	[0.32; 1.22]	52.6%
ARMYDA-5	1	204	0	205		11.11	[0.02; 7216.67]	0.2%
CREDO	0	1053	4	1063		0.02	[0.00; 13.01]	9.7%
CURE PCI	14	1313	13	1345		1.10	[0.52; 2.36]	30.3%
**Fixed effect model**		**5318**		**5330**		**0.74**	**[0.46; 1.17]**	**100.0%**

Heterogeneity: $I^2 = 0\%$, $p = 0.54$

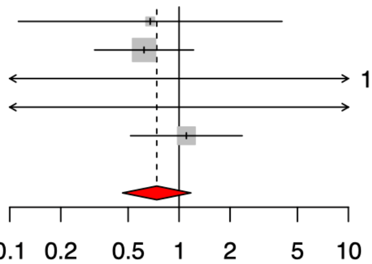

D

30-day Major Bleeding	Early n	Early N	Late n	Late N	Odds Ratio	OR	95% CI	Weight
DUBIUS	14	711	12	721		1.19	[0.55; 2.58]	6.5%
ISAR-REACT 5	40	1179	28	1186		1.45	[0.89; 2.37]	15.1%
ACCOAST	100	2037	39	1996		2.59	[1.78; 3.77]	21.0%
ARMYDA-5	16	204	11	205		1.50	[0.68; 3.32]	5.7%
CREDO	83	1053	62	1063		1.38	[0.98; 1.94]	31.9%
CURE PCI	34	1313	29	1345		1.21	[0.73; 1.99]	15.6%
Bonello et al	7	106	8	107		0.88	[0.31; 2.51]	4.2%
**Fixed effect model**		**6603**		**6623**		**1.59**	**[1.32; 1.92]**	**100.0%**

Heterogeneity: $I^2 = 41\%$, $p = 0.12$

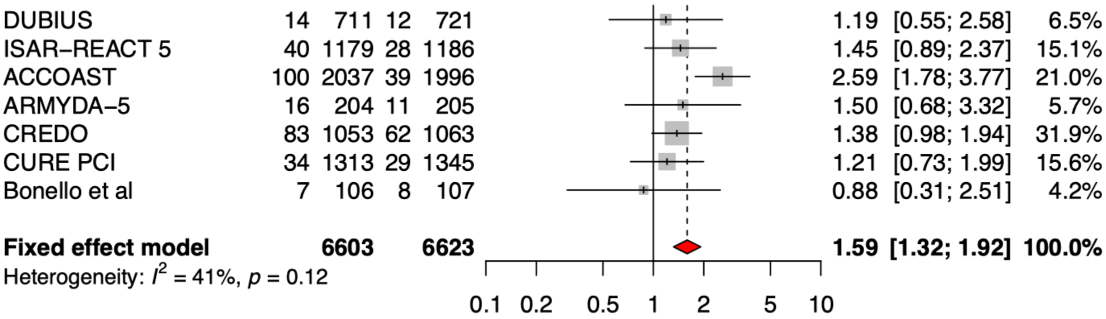

© 2021 Dawson LP et al. *JAMA Network Open.*

eFigure 4. Sensitivity Analysis Excluding PCI CURE From P2Y12 Inhibitor Subgroup Analysis

30–day MACE	Early n	Early N	Late n	Late N	OR	95% CI	Weight
type = Ticagrelor							
DUBIUS	24	711	21	721	1.16	[0.64; 2.11]	9.0%
ISAR–REACT 5	39	1179	30	1186	1.32	[0.81; 2.14]	13.2%
Bonello et al	4	106	5	107	0.80	[0.21; 3.06]	1.9%
Random effects model		1996		2014	1.21	[0.85; 1.74]	24.1%
Prediction interval	.	.	.	.		[0.12; 12.63]	---
Heterogeneity: $I^2 = 0\%$, $p = 0.78$							
type = Prasugrel							
ACCOAST	157	2037	146	1996	1.06	[0.84; 1.34]	43.6%
Random effects model		2037		1996	1.06	[0.84; 1.34]	43.6%
Prediction interval	.	.	.	.			---
Heterogeneity: not applicable							
type = Clopidogrel							
ARMYDA–5	21	204	18	205	1.19	[0.62; 2.31]	7.4%
CREDO	65	1053	83	1063	0.78	[0.55; 1.09]	24.9%
Random effects model		1257		1268	0.87	[0.60; 1.27]	32.3%
Prediction interval	.	.	.	.			---
Heterogeneity: $I^2 = 22\%$, $p = 0.26$							
**Random effects model**		**5290**		**5278**	**1.02**	**[0.85; 1.23]**	**100.0%**
**Prediction interval**						**[0.73; 1.43]**	
Heterogeneity: $I^2 = 0\%$, $p = 0.52$							

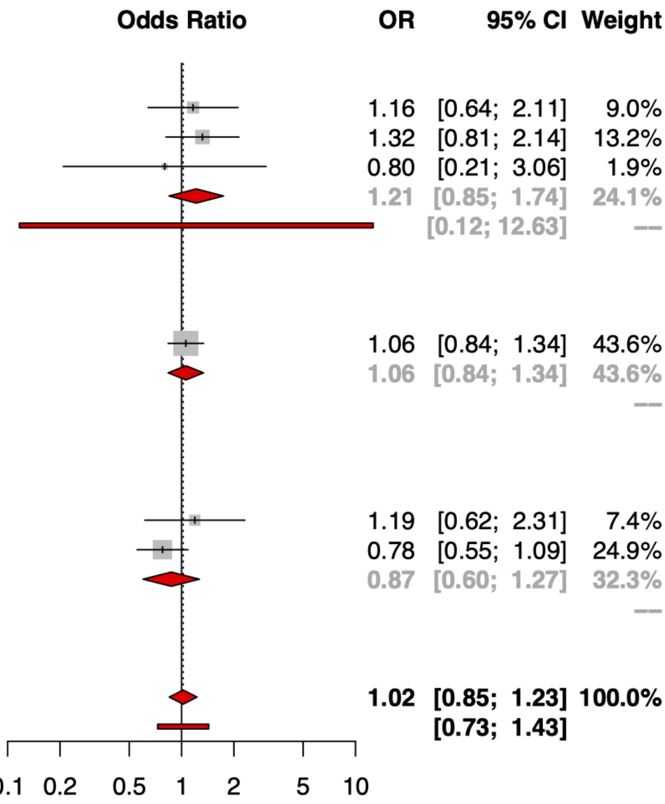

© 2021 Dawson LP et al. *JAMA Network Open.*

eFigure 5. Subgroup Analyses for the Primary Safety End Point (Bleeding)
Stratified by (A) P2Y12 inhibitor used for pre-treatment, (B) revascularisation strategy, (C) arterial access site.

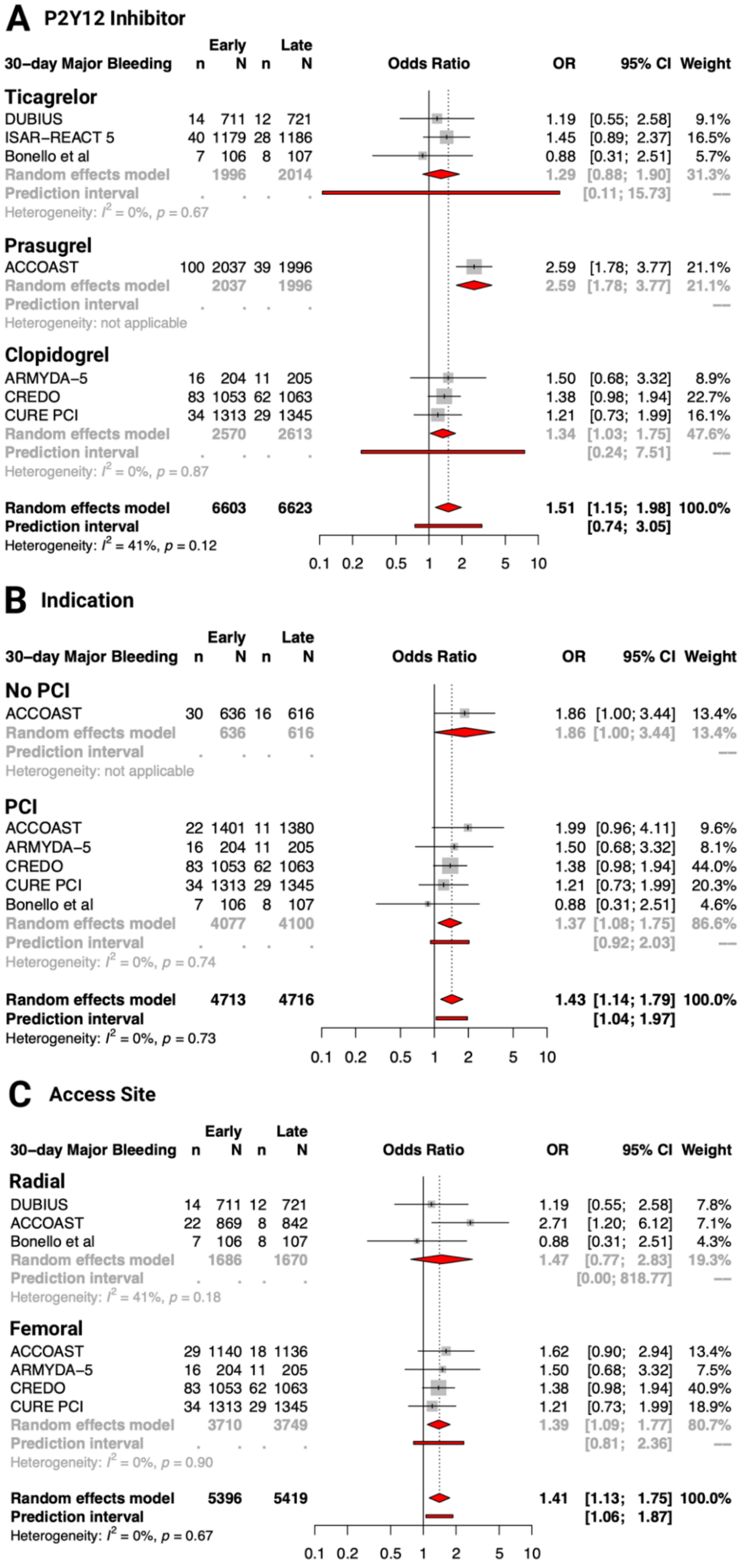

© 2021 Dawson LP et al. *JAMA Network Open.*

eFigure 6. Leave-One-Out Sensitivity Analyses

eFigure 6a. Leave-one-out sensitivity analysis for 30-day MACE

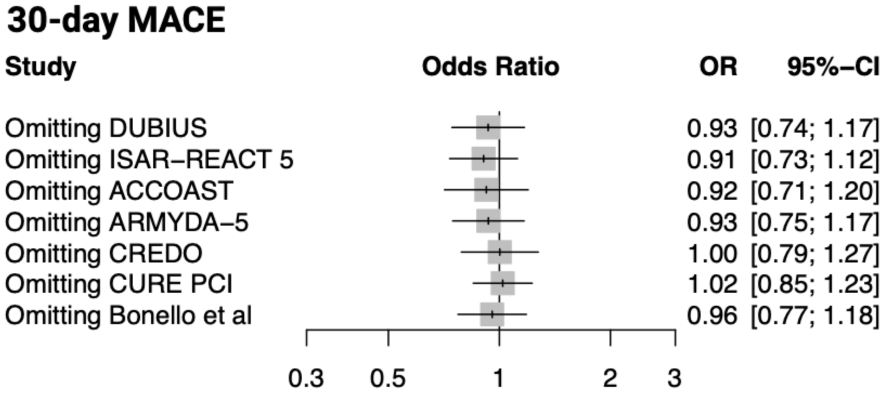

eFigure 6b. Leave-one-out sensitivity analysis for 30-day Major Bleeding

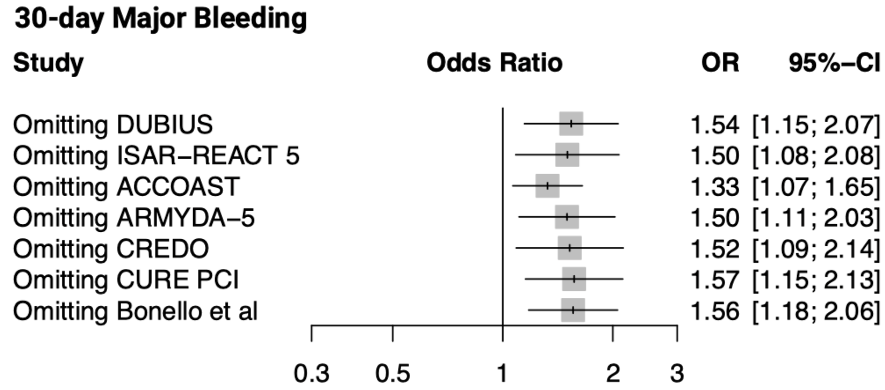

eFigure 6c. Leave-one-out sensitivity analysis for 30-day Cardiovascular Mortality

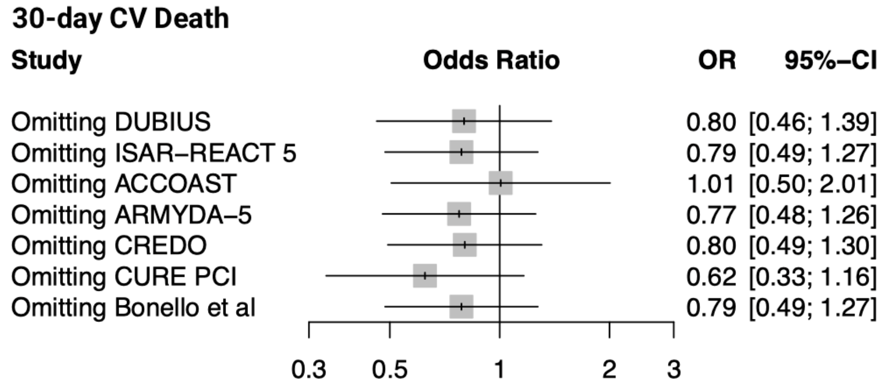

eFigure 6d. Leave-one-out sensitivity analysis for 30-day Myocardial Infarction

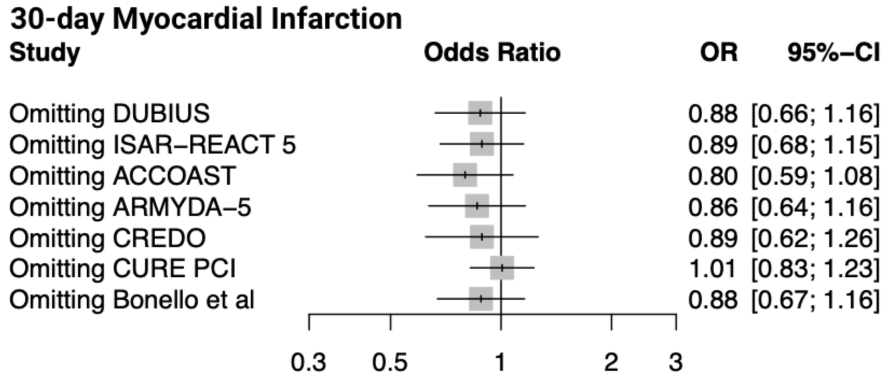

© 2021 Dawson LP et al. *JAMA Network Open.*

eFigure 7. Metaregression Assessing Timing of Pretreatment Loading Dose

eFigure 7a. Meta-regression assessing impact of P2Y12 inhibitor administration timing on 30-day MACE in all studies (meta-regression coefficient 0.0028, p=0.0629)

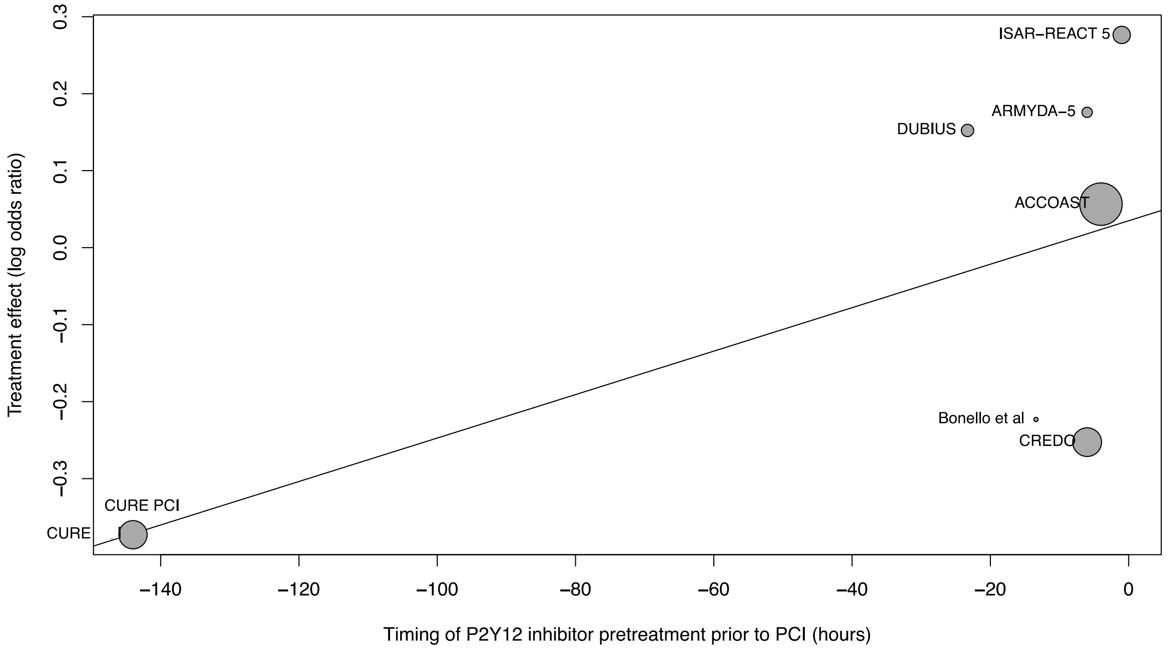

eFigure 7b. Meta-regression assessing impact of P2Y12 inhibitor administration timing on 30-day MACE with CURE PCI trial excluded to more reflect a contemporary early invasive strategy (meta-regression coefficient 0.0006, p=0.0362)

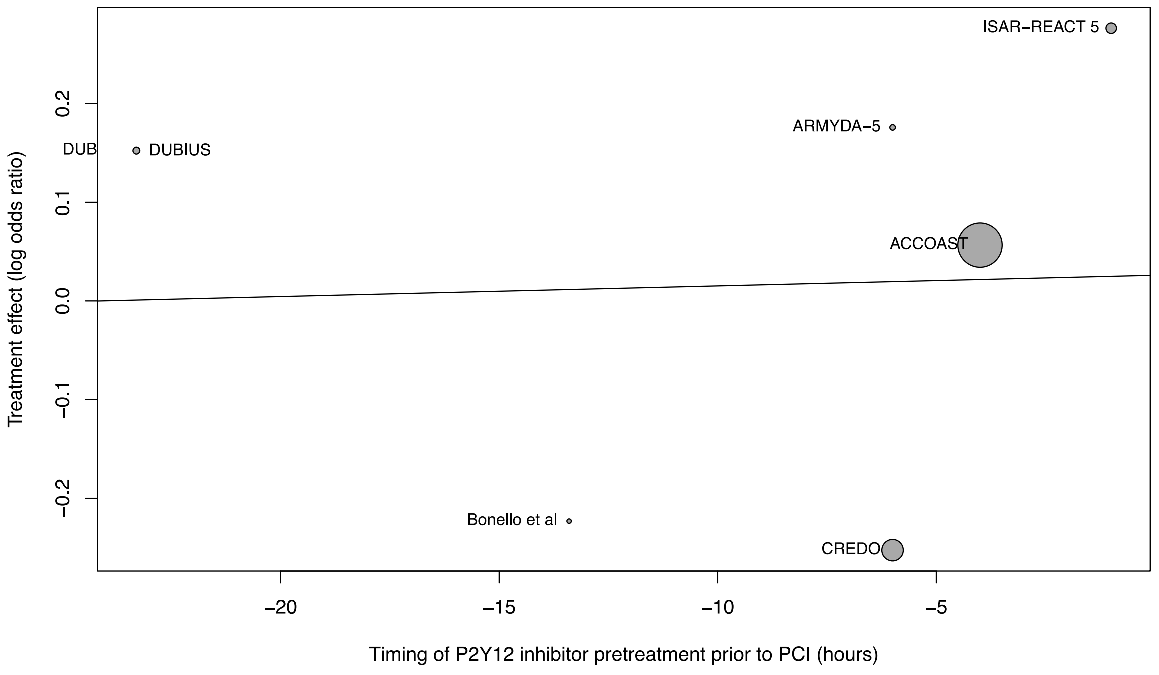

© 2021 Dawson LP et al. *JAMA Network Open.*

eFigure 7c. Meta-regression assessing impact of P2Y12 inhibitor administration timing on 30-day major bleeding in all studies (meta-regression coefficient 0.0022, p=0.43)

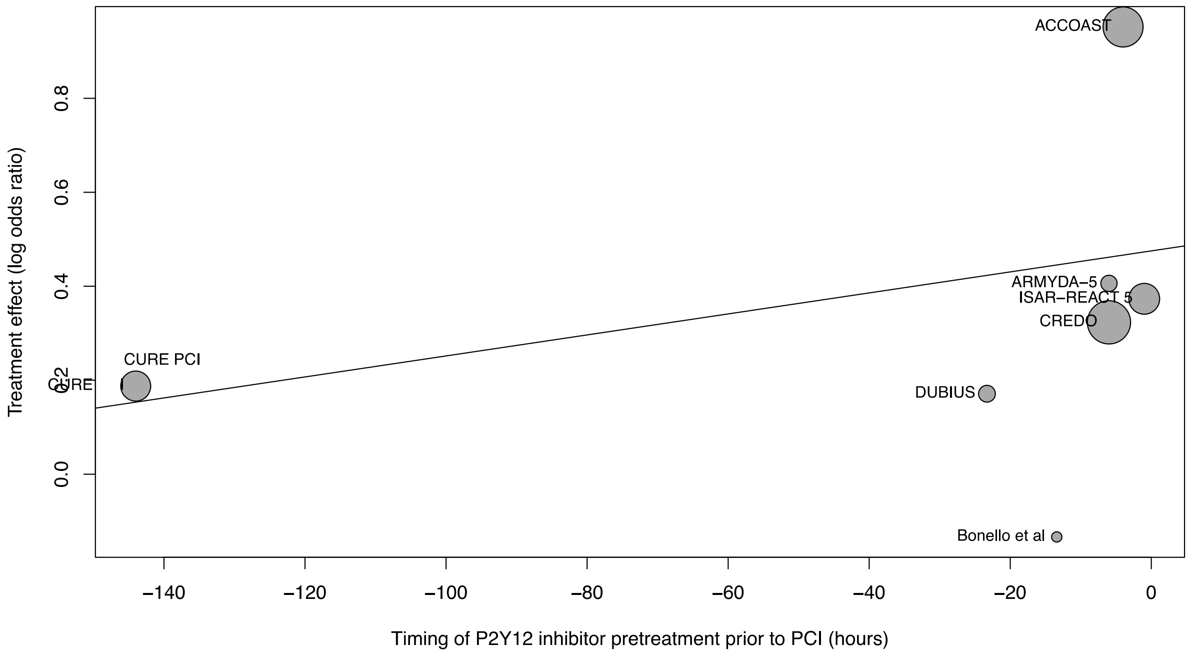

eFigure 7d. Meta-regression assessing impact of P2Y12 inhibitor administration timing on 30-day major bleeding with CURE PCI trial excluded to more reflect a contemporary early invasive strategy (meta-regression coefficient 0.0240, p=0.3159)

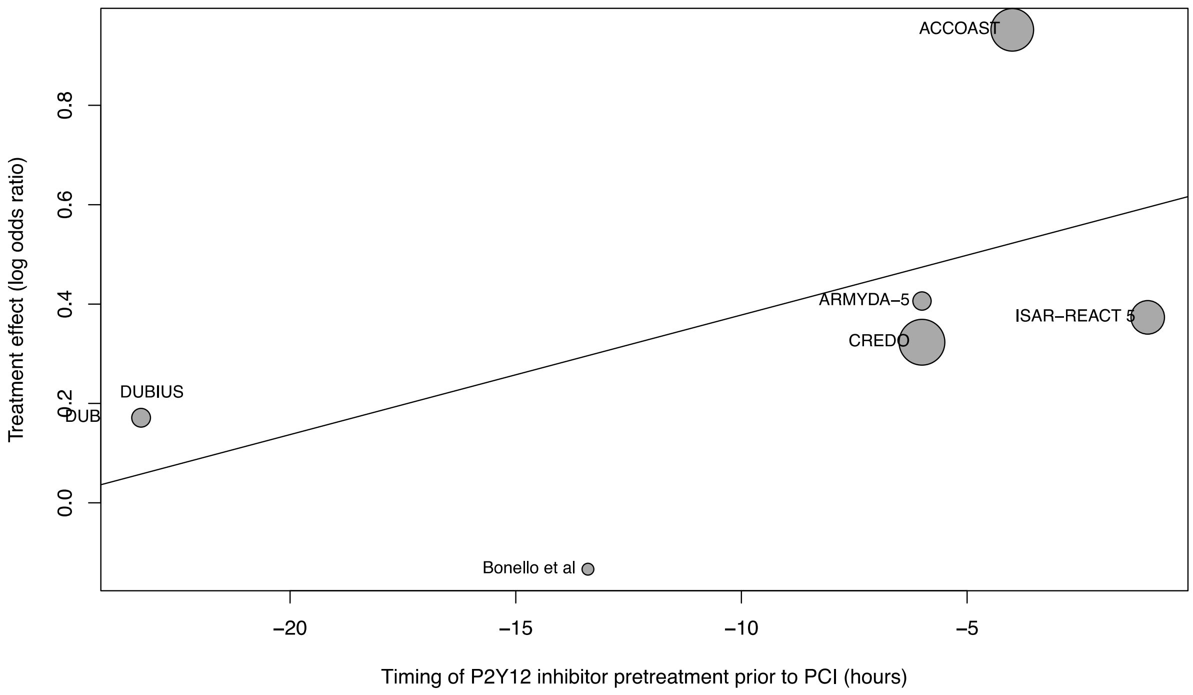

© 2021 Dawson LP et al. *JAMA Network Open.*

eReferences.

1. Mehta SR, Yusuf S, Peters RJG, Bertrand ME, Lewis BS, Natarajan MK, et al. Effects of pretreatment with clopidogrel and aspirin followed by long-term therapy in patients undergoing percutaneous coronary intervention: The pci-cure study. *Lancet*. 2001;358:527-533
2. Steinhubl SR, Berger PB, Mann JT, 3rd, Fry ET, DeLago A, Wilmer C, et al. Early and sustained dual oral antiplatelet therapy following percutaneous coronary intervention: A randomized controlled trial. *JAMA*. 2002;288:2411-2420
3. Di Sciascio G, Patti G, Pasceri V, Gatto L, Colonna G, Montinaro A, et al. Effectiveness of in-laboratory high-dose clopidogrel loading versus routine pre-load in patients undergoing percutaneous coronary intervention: Results of the armyda-5 preload (antiplatelet therapy for reduction of myocardial damage during angioplasty) randomized trial. *Journal of the American College of Cardiology*. 2010;56:550-557
4. Montalescot G, Collet JP, Ecollan P, Bolognese L, Ten Berg J, Dudek D, et al. Effect of prasugrel pre-treatment strategy in patients undergoing percutaneous coronary intervention for nstemi: The accoast-pci study. *Journal of the American College of Cardiology*. 2014;64:2563-2571
5. Montalescot G, Bolognese L, Dudek D, Goldstein P, Hamm C, Tanguay JF, et al. Pretreatment with prasugrel in non-st-segment elevation acute coronary syndromes. *New England Journal of Medicine*. 2013;369:999-1010
6. Bonello L, Laine M, Cluzel M, Frere C, Mancini J, Hasan A, et al. Comparison of ticagrelor versus prasugrel to prevent periprocedural myonecrosis in acute coronary syndromes. *American Journal of Cardiology*. 2015;116:339-343
7. Valina C, Neumann FJ, Menichelli M, Mayer K, Wohrle J, Bernlochner I, et al. Ticagrelor or prasugrel in patients with non-st-segment elevation acute coronary syndromes. *Journal of the American College of Cardiology*. 2020;76:2436-2446
8. Tarantini G, Mojoli M, Varbella F, Caporale R, Rigattieri S, Ando G, et al. Timing of oral p2y12 inhibitor administration in non-st elevation acute coronary syndrome. *Journal of the American College of Cardiology*. 2020
9. Dworeck C, Redfors B, Angerås O, Haraldsson I, Odenstedt J, Ioanes D, et al. Association of pretreatment with p2y12 receptor antagonists preceding percutaneous coronary intervention in non-st-segment elevation acute coronary syndromes with outcomes. *JAMA Netw Open*. 2020;3:e2018735
10. Verdoia M, Pergolini P, Barbieri L, Rolla R, Nardin M, Negro F, et al. Impact of pre-procedural dual antiplatelet therapy on periprocedural myocardial infarction in patients undergoing percutaneous coronary interventions with adjunctive tirofiban. *Thrombosis Research*. 2018;164:17-23
11. Sukul D, Seth M, Dixon SR, Khandelwal A, Lalonde TA, Gurm HS. Contemporary trends and outcomes associated with the preprocedural use of oral p2y12 inhibitors in patients undergoing percutaneous coronary intervention: Insights from the blue cross blue shield of michigan cardiovascular consortium (bmc2). *Journal of Invasive Cardiology*. 2017;29:340-351
12. Yudi MB, Eccleston D, Andrianopoulos N, Farouque O, Duffy SJ, Brennan A, et al. Pre-treatment with dual antiplatelet therapy in patients with non-st-segment elevation acute coronary syndromes undergoing percutaneous coronary intervention. *Internal Medicine Journal*. 2015;45:1032-1037

© 2021 Dawson LP et al. *JAMA Network Open.*

13. Ikegami Y, Kohsaka S, Miyata H, Ueda I, Fuse J, Sakamoto M, et al. Outcomes of percutaneous coronary intervention performed with or without preprocedural dual antiplatelet therapy. *Circulation Journal*. 2015;79:2598-2607
14. Almendro-Delia M, Gonzalez-Torres L, Garcia-Alcantara A, Reina-Toral A, Sanchez JAA, Yanez JCR, et al. Prognostic impact of clopidogrel pretreatment in patients with acute coronary syndrome managed invasively. *Am. J. Cardiol.* 2015;115:1019-1026
15. Wang TY, White JA, Tricoci P, Giugliano RP, Zeymer U, Harrington RA, et al. Upstream clopidogrel use and the efficacy and safety of early eptifibatide treatment in patients with acute coronary syndrome: An analysis from the early glycoprotein iib/iiia inhibition in patients with non-st-segment elevation acute coronary syndrome (early acs) trial. *Circulation*. 2011;123:722-730
16. Feldman DN, Kim LK, Minutello RM, Bergman G, Moussa I, Wong SC. Can clopidogrel loading (600mg) be administered <2 hours pre-pci in patients presenting with acute coronary syndromes? *Journal of the American College of Cardiology*. 2010;55 (10 SUPPL 1):A206.E1942
17. Lincoff AM, Steinhubl SR, Manoukian SV, Chew D, Pollack Jr CV, Feit F, et al. Influence of timing of clopidogrel treatment on the efficacy and safety of bivalirudin in patients with non-st-segment elevation acute coronary syndromes undergoing percutaneous coronary intervention. An analysis of the acuity (acute catheterization and urgent intervention triage strategy) trial. *JACC: Cardiovascular Interventions*. 2008;1:639-648
18. Szük T, Gyöngyösi M, Homorodi N, Kristóf E, Király C, Edes IF, et al. Effect of timing of clopidogrel administration on 30-day clinical outcomes: 300-mg loading dose immediately after coronary stenting versus pretreatment 6 to 24 hours before stenting in a large unselected patient cohort. *Am Heart J*. 2007;153:289-295
19. Dery JP, Campbell ME, Mathias J, Pieper KS, Harrington RA, Madan M, et al. Complementary effects of thienopyridine pretreatment and platelet glycoprotein iib/iiia integrin blockade with eptifibatide in coronary stent intervention; results from the esprit trial. *Catheter Cardiovasc Interv*. 2007;70:43-50
20. Chan AW, Moliterno DJ, Berger PB, Stone GW, DiBattiste PM, Yakubov SL, et al. Triple antiplatelet therapy during percutaneous coronary intervention is associated with improved outcomes including one-year survival: Results from the do tirofiban and reoprogive similar efficacy outcome trial (target). *J Am Coll Cardiol*. 2003;42:1188-1195
21. Assali AR, Salloum J, Sdringola S, Moustapha A, Ghani M, Hale S, et al. Effects of clopidogrel pretreatment before percutaneous coronary intervention in patients treated with glycoprotein iib/iiia inhibitors (abciximab or tirofiban). *Am J Cardiol*. 2001;88:884-886, a886
